# Exposure to UV radiance predicts repeated evolution of concealed black skin in birds

**DOI:** 10.1038/s41467-020-15894-6

**Published:** 2020-05-15

**Authors:** Michaël P. J. Nicolaï, Matthew D. Shawkey, Sara Porchetta, Ruben Claus, Liliana D’Alba

**Affiliations:** 1 Biology Department, Evolution and Optics of Nanostructures Group, Ghent University, Ledeganckstraat 35, 9000 Ghent, Belgium; 2 Department of Recent Vertebrates, Royal Belgian Institute of Natural Sciences, Leuven, Belgium; 3 Department of Earth and Environmental Sciences, KULeuven, Leuven, Belgium; 4 von Karman Institute for Fluid Dynamics, Sint-Genesius-Rode, Belgium

**Keywords:** Biogeography, Evolutionary ecology, Phylogenetics

## Abstract

Plumage is among the most well-studied components of integumentary colouration. However, plumage conceals most skin in birds, and as a result the presence, evolution and function of skin colour remains unexplored. Here we show, using a database of 2259 species encompassing >99% of bird genera, that melanin-rich, black skin is found in a small but sizeable percentage (~5%) of birds, and that it evolved over 100 times. The spatial distribution of black skin follows Gloger’s rule, which states that pigmentation of endothermic animals increases towards the equator. Furthermore, most black-skinned birds inhabit high irradiation regions, and tend to be bald and/or have white feathers. Thus, taken together, our results suggest that melanin-rich, black skin helps to protect birds against ultraviolet irradiation. More generally, our results illustrate that feathered skin colour varies taxonomically, ontogenetically and temporally, providing an additional dimension for avian colour research.

## Abstract (web summary)

In contrast to bird plumage, little is known about the evolution of bird skin color. Here, Nicolaï et al. find that black skin has evolved over 100 times in birds and is associated with baldness and/or white feathers as well as with high irradiation habitats, suggesting a role in UV protection.

## Introduction

**Fig. 1:**
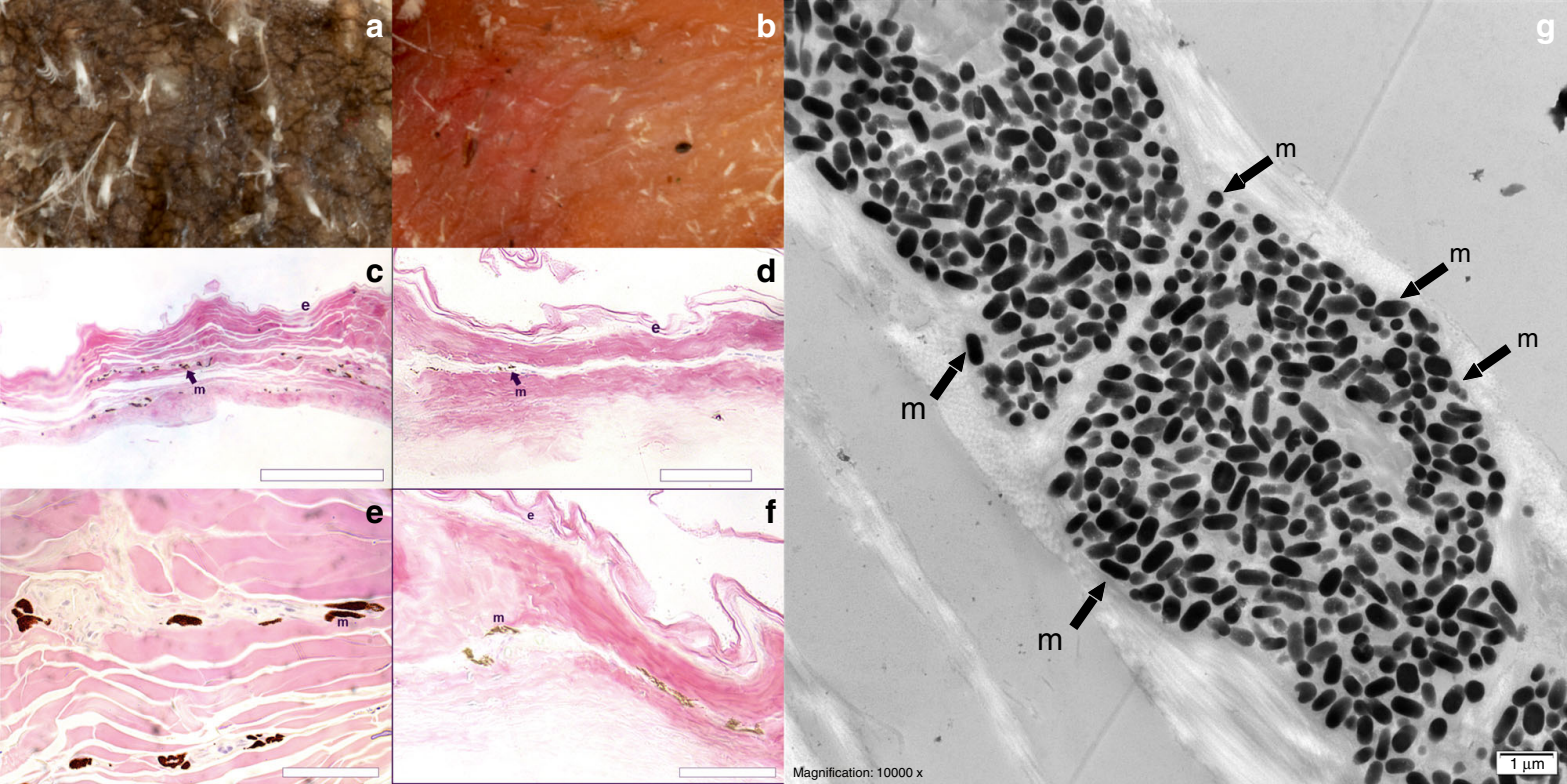
Differences in skin colour of (**a**) Morus bassanus (black) and (**b**) Garrulus glandarius (red) are due to differences in melanin concentration as seen under light microscopy for (**c**, **e**) M. *bassanus* and (**d**, **f**) for *G. glandarius*. Scale bars represent 50 μm (**c**, **d**), 30 µm (**e**, **f**) and 1 µm (**g**). e stands for epidermis, m for melanin. While melanin is present in both tissues, concentrations appear much higher in black skin. **g** Detail of a melanophore containing individual melanosomes (few examples indicated by an m) from *M. bassanus* (scale bar 1 µm).

The immense variety of colours in birds has fascinated scientists since Darwin^1^ and Newton^2^, and in the past twenty years has received considerable attention in fields as diverse as ecology and optics^3,4^. This research has focused primarily on plumage colouration, with the exceptions of a few studies on some of the structurally-coloured blue, orange or yellow colours of ramphotheca, podotheca and other exposed (unfeathered) parts of the skin^5,6^. Colouration and associated evolutionary mechanisms of black, unexposed (feathered) skin are practically unexplored^7^. The presence of black bird skin (Fig. 1 and Supplementary Figs. 1–4) has previously been documented, but mostly anecdotally and out of an evolutionary context, in a handful of bird species^5–13^.

Black skin in vertebrates is, as far as we are aware, always produced by the deposition of (eu)melanin, the most ubiquitous pigment among animals^14^. Light and transmission electron microscopy confirmed that black skin in birds also has more melanin (deposited in oblate dark organelles called melanosomes) than non-black skin (Fig. 1 and Supplementary Fig. 2). Melanin pigments have a unique combination of properties such as a high refractive index (~1.8–2.0) and broad light absorption that is particularly strong in the UV range^14^, which has led to a range of adaptive functions^15–17^.

First, melanin may protect skin against DNA-damaging UV irradiation (300–400 nm) by reducing UV transmission to the skin, as suggested for melanin in amphibian skin^18^, lizard skin^19^, bird feathers^20^, and human skin^16,21^. However, the effectiveness of melanin-based UV protection has been questioned^22–24^, and its relevance depends on the transmission of light by the overlying plumage. Indeed, darker (brown, black, and yellow–green) feathers transmit up to 40% less UV light than lighter (white, orange, and yellow) feathers^25,26^, most likely due to higher light absorption by melanin. Additionally, UV exposure varies between different habitats, with open habitats receiving more UV light. As such, protection from UV irradiation is achieved through the interplay between physiological and ecological parameters that contribute to differences in UV exposure.

In addition to protecting against UV, skin pigmentation is also involved in modulating body temperature by reflecting or absorbing solar radiation, or by enhancing water evaporation^15,27^. Both theoretical and experimental evidence suggests that dark feathers gain more heat than white feathers, and thus dark and light skin colour may follow the same pattern^15,23,26^. One previous study showed that the black dorsal skin patch of the roadrunner (*Geococcyx californianus*), when exposed during sunbathing, passively rewarmed the bird after cold nights^12^. Faster heat gain would be most beneficial to species inhabiting cold and/or dry regions, and in particular for small species with high surface to volume ratios^28^.

Melanin-rich, black skin may also confer antimicrobial protection. Previous studies have shown that melanocytes, the cells that contain melanin have antimicrobial properties, and prevent microbial feather degradation^17,29–32^. As in feathers, black skin might thus reduce bacterial and fungal growth on dermis and epidermis. This would be particularly advantageous in environments with increased infection rate (e.g., hot and moist climates) as well as in species with frequent social interactions.

Although unlikely, given its obscured position below feathers, skin colour might also be used in communication, to signal various aspects of physiological condition or social status in bird, and as a result may be under sexual selection^9^. Previous studies have suggested that melanized feathers might indicate male quality, and even small mutations produce different colour morphs^33,34^. Should sexual selection drive the evolution of black skin, we predict that sexual preference would result in discernible sexual dichromatism.

At least three of these hypotheses (thermoregulation, antimicrobial-protection, or photo-protection) are implicated as mechanisms behind Gloger’s rule, an ecogeographic rule that states that melanin in mammals and birds increases towards the equator and has been supported for feathers, fur, and human skin^16,35–38^. Whether this rule holds true for concealed skin, whether mammalian or avian, is unknown.

To understand how the distribution of black skin across birds relates to biogeographical, ecological and social conditions we use a phylogenetic comparative framework, together with distribution modelling. We find that black skin is more common towards the equator, following Gloger’s rule, and that black skin evolved convergently over 100 times, in different bird clades. While multiple mechanisms have been proposed for this rule, the combination of the association of black skin with white or no plumage, as well as high UV irradiance, suggest that it is best explained in birds by a photoprotective function. In line with this function are the preliminary observations that skin colour changes both seasonally and ontogenetically, being darker in times when plumage coverage, and thus feather photoprotection, is low.

## Results and discussion

### Evolution of black skin in birds

**Fig. 2:**
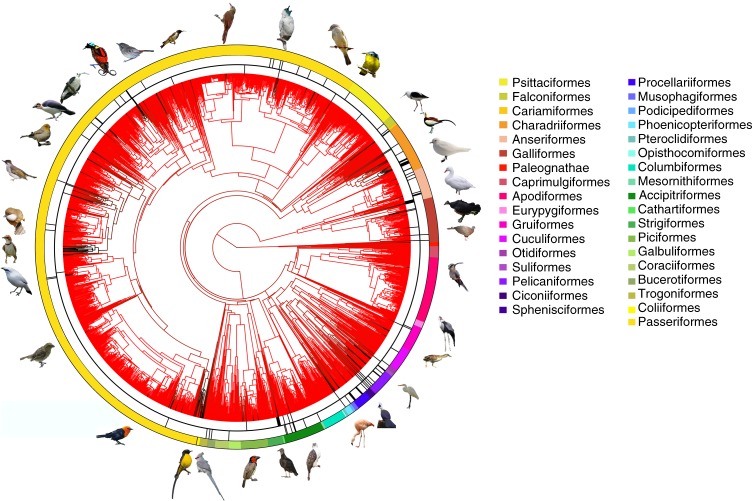
Ancestral state estimation for black skin of the heads of male birds. Branches of the phylogenetic tree are black when the reconstructed skin colour was black, and red when this was non-black. Black bars in the first circle represent species that are bald, while black bars in second circle represent species that have white feathers. All photographs were edited such that the background was removed. Photographs were taken by U.S. Fish & Wildlife Service (*Telespiza cantans*), Kenvanportbc (*Amblyramphus holosericeus*) and Adrian Pingstone (*Coscoroba coscoroba*) in the public domain, by Hector Bottai (*Hylexetastes perrotii*, *Hapalocrex flaviventer*) and Charles J Sharp (*Ardea alba*) under CC BY-SA 4.0 (https://creativecommons.org/licences/by-sa/4.0/), by Thai National Parks (*Hemixos flavala*), birdphotos.com (*Bugeranus carunculatus*) and Dubi Shapiro (*Neodrepanis hypoxantha*) under CC BY SA 3.0 (https://creativecommons.org/licences/by-sa/4.0/), by Flickr-users under CC BY 2.0 Bernard DUPONT (*Lybius torquatus*), Lip Kee (*Camptostoma obsoletum*), Francesco Veronesi (*Hedydipna platura*), Ben Tavener (*Procnias nudicollis*), Michael Andersen (*Picathartes gymnocephalus*), Frank Vassen (*Falculea palliata*), jquental (*Anopetia gounellei*), Alastair Rae (*Malcorus pectoralis*), Brian Jelonek (*Tauraco leucolophus*) and Alan D. Wilson (*Auriparus flaviceps*) under CC BY SA 2.5 (https://creativecommons.org/licences/by-sa/2.5/), by wiki-users Ariefrahman (*Macrocephalon maleo*), Mdk572 (*Sugomel niger*), Peripitus (*Hylacola cautus*), Prateik Kulkarni (*Ianthocincla albogularis*), Dasari. Vijay (*Hydrophasianus chirurgus*) under CC BY SA 4.0 (https://creativecommons.org/licences/by-sa/4.0/), Doug Janson (*Urocolius macrourus*), Helenabella (C*ladorhynchus leucocephalus*), Mdf (*Coragyps atratus*), Tragopan (*Phoenicopterus chilensis*), DickDaniels (*Argusianus argus*), Serhanoksay (*Cicinnurus respublica*) under CC BY SA 3.0 (https://creativecommons.org/licences/by-sa/3.0/) and scorpious18 (*Pithecophaga jefferyi*), jomilo75 (*Pagophila eburnean*), and Jcwf (*Leucopsar rothschildi*) under CC BY SA 2.0 (https://creativecommons.org/licences/by/2.0/).

Our analysis of museum specimens of 2247 species from all families and >99% of bird genera indicates that black skin is a taxonomically widespread trait (Fig. 2). Black skin on the head is present in at least 138 genera (6%) from 59 families (23%), while black skin of the venter is present in 11 genera (0.5%) from 6 families (2%). Furthermore, ancestral state estimation showed that black skin on the head evolved independently at least 148 times (118 times in the gene-only phylogeny, Supplementary Fig. 5), but was lost more often than it evolved (Fig. 2). This widespread independent evolution is reflected in the absence of phylogenetic signal (Fritz and Purvis’ *D* > 0.98, *P*(Brownian motion) = 0, *P*(random) = 0, where *D* = 0 indicates the trait is as conserved as expected under BM, where a value of *D* = 1 indicates randomness) in head skin colour. Black head skin was lost 15 times more often than it was gained (rate_gain_ = 0.005, rate_loss_ = 0.071; *p*-value = 0; chi-squared; independent from phylogenetic tree used). Low evolutionary rates, and highly localised presence on the body suggest that black skin might be evolutionary costly and might only evolve when it serves a particular function.

### Black skin is best explained by exposure to UV radiance

**Fig. 3:**
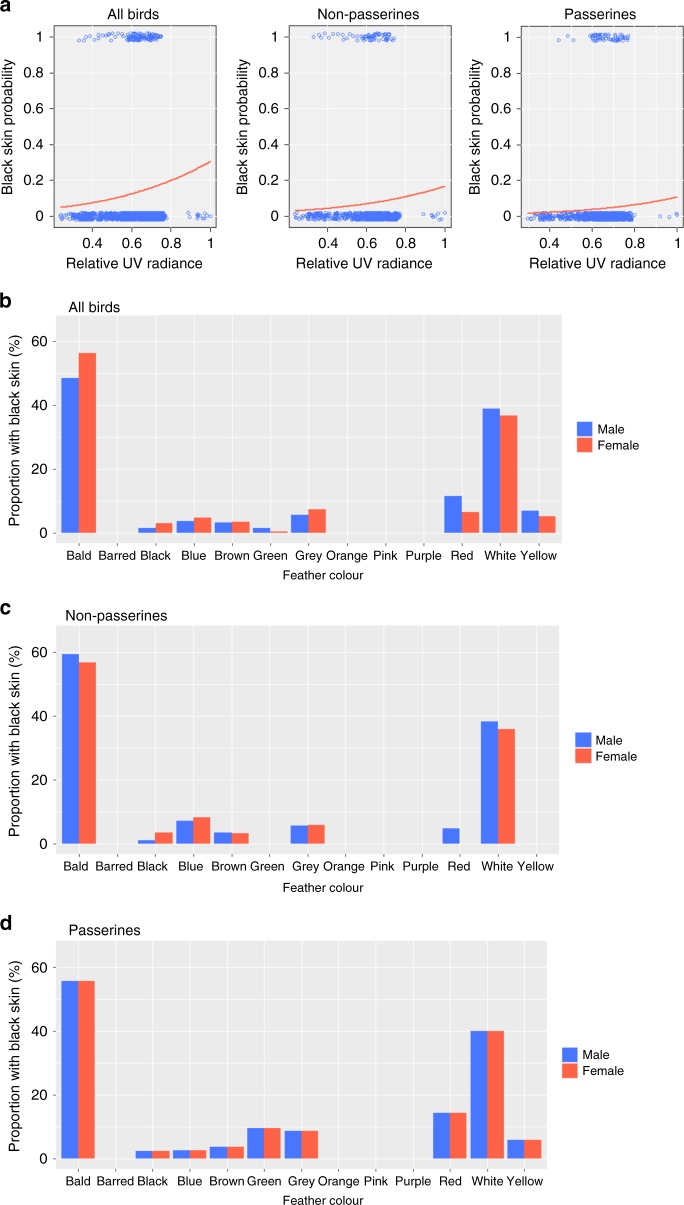
Black skin in function of UV radiance and feather colour. **a** Black skin probability in function of relative UV radiance (UV radiance/max UV radiance) for all birds, non-passerines and passerines (data shown for female dataset only). Observed proportion of black skin relative to non-black skin in function of feather colour for all birds (**b**), non-passerines (**c**) and passerines (**d**) (Supplementary Table 8).

To test whether black skin could function as protection against harmful UV, we investigated the association between black skin and exposure to UV radiance, i.e., bald or lightly coloured birds in high UV zones. Feather colour and UV radiance were best at predicting black skin colour except for non-passerines (Supplementary Table 1). Species that were bald (coefficient estimate > 1.37; *p*-values between <0.001 and 0.38, except for non-passerine analyses where pattern present but not significant), or had white plumage (coefficient estimate > 0.59; *p*-values between <0.001 and 0.38, except for passerine analyses where the pattern was present but not significant) had a high probability of having black skin, as did species that occured in high UV radiance zones regardless of feather colour (coefficient estimate always positive except for male passerine analyses where negative; *p*-values between <0.001 and 0.30) (Supplementary Tables 2–4) (Figs. 2, 3). All other (non-white) feather colours were strictly negatively associated with black skin to different degrees of significance (Supplementary Tables 2–4). Unexpectedly, an analysis that incorporated habitat type as an exposure proxy, i.e., being either open or closed, did not perform better, perhaps because micro-habitat might be more relevant for UV radiance exposure in highly mobile birds. Feather density also likely influences UV protection, and our finding that black skin is mostly absent ventrally, while present on the head (where exposure to UV is high and plumage density is low^39^), further supports the UV protection hypothesis. This might explain why the effect of UV and feather colour is less apparent in more densely feathered non-passerines. Thus, three factors (thin or no plumage, light plumage and high UV irradiation) predicted under a UV protection hypothesis are linked with black skin in birds. Interestingly, this pattern holds true for extinct birds: black skin was present in the hoopoe starling (*Fregilupus varius*) and reconstructed for the dodo (*Raphus cucullatus*), both species of light (or no) plumage living in high irradiation zones (Supplementary Table 9).

In contrast, we found little evidence for the thermal regulation hypothesis when using temperature and rainfall as proxies of thermal regulation (Supplementary Table 1). For example, heat loss is disproportionally larger in smaller birds, but our data does not show the expected negative correlation between black skin and mass, as predicted if black skin contributes to heat gain (Supplementary Tables 2–4). Nonetheless, the effect of precipitation is more complex with total precipitation generally being negatively correlated, as expected, to black skin (coefficient estimate between −1.84 and 0.23; *p*-values between 0.01 and 0.75; Supplementary Tables 2–4), although this could be the result of a similar effect on biodiversity in general.

We also found little support for a bacterial protection hypothesis. Colonial birds have a weakly (coefficient estimate between 0.04–0.42; *p*-values between 0.04–0.76; Supplementary Tables 2–4) increased probability of having black skin. Other abiotic factors linked to higher infection rates (Supplementary Tables 2–4) had variable responses on black skin (Precipitation: coefficient estimates between −1.17 and −0.41; *p*-values between 0.01 and 0.26; Maximum temperature: coefficient estimates between −0.04 and 1.17; *p*-values between 0.01 and 0.35).

Surprisingly, we found a lower probability of black skin in sexually dimorphic species with varying degrees of significance (coefficient estimates between −1.14 and −0.31,* p*-values between 0.01 and 0.67; Supplementary Tables 5–7), except for male non-passerines where the effect was positive but insignificant (coefficient estimates between 0.10 and 0.23  * p*-values between 0.42 and 0.75; Supplementary Tables 5–7). Nonetheless, sexual selection cannot be completely ruled out. Other non-studied factors (e.g., mutual sexual selection) might be important, as supported by the association of black skin with baldness, which suggests a possible use of black head colour as an ornament.

### Black skin follows Gloger’s rule

**Fig. 4:**
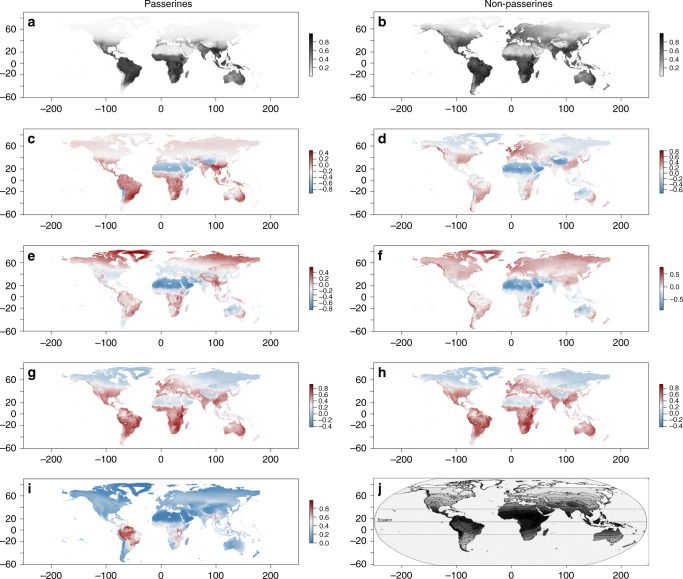
Probability of black skin occurrence estimated by MAXENT^56^ using solar radiation (kJm-2day-1), average precipitation (mm), and average maximum temperature (°C) as predictor values in passerines (A) and non-passerines (B). Additional panes reflect the difference of the predicted black skin probabilities in relation to UV radiation in passerines (**c**) and non-passerines (**d**); The difference of predicted probabilities and relative maximum temperature (maximum temperature/maximal maximum temperature value) in passerines (**e**) and non-passerines (**f**); The difference of predicted probabilities and relative precipitation (precipitation/maximal precipitation value) in passerines (**g**) and non-passerines (**h**); The relative bird biodiversity (bird biodiversity/maximal bird biodiversity) (**i**) and the distribution of black skin colour in humans (**j**) (Adapted from Chaplin^40^). The darkness gradients in **a**, **b** and **j** indicate the probability of black skin being present: high probability regions are dark, low probability regions are white. In **b**–**d** values near 0 (white) indicate a good fit between predictor value and black skin probability while non-zero values indicate increasing tendencies to a mismatch between predictor and predictor indicating that another variable contributes more to the black skin prediction model. All climatic variables were yearly averaged over a span of 30 years.

Skin melanization in birds increases towards lower latitudes, particularly when considering their breeding range only (*p*-values < 0.03 in all birds and passerines, but not significant and reversed in non-passerine analyses) (Supplementary Tables 2–4). This is consistent with the predictions of Gloger’s rule, at least for passerines. When using occurrence localities, instead of average breeding range, Gloger’s rule is supported for both passerines and non-passerines (Fig. 4a, b). Although black skin density is highest in the tropics, where species diversity in general is higher, black skin occurred more often than predicted by bird diversity alone (Fig. 4i). As such, bird diversity is not a particularly good predictor of black skin. The results from our niche prediction model, which incorporates total distribution ranges, do, however, suggest that for passerines UV radiation is the best predictor of black skin (Supplementary Figure 6). For non-passerine birds, maximum temperature explains the data best. Nonetheless, even for non-passerines, there are regions of low or high UV radiance that correspond, respectively, to low or high black skin density, even in zones where bird diversity follows an opposite pattern (e.g., in South America) (Fig. 4c, d). The disparity between the niche model and UV radiance in passerines might thus be the result of autocorrelation between (UV) radiance and temperature. Furthermore, humans have a similar distribution of black skin as birds and analyses show a strong effect of radiance, providing further support for a protective function against UV (Fig. 4j)^40^.

### Black skin is not the result of preservation differences

One final mechanism explaining differences in colouration is through differences in preservation. Melanization does indeed occur as a consequence of trauma in skins of fruits and humans^41^ and we did occasionally observe this in bird specimens. However, this was distinct from naturally black skin, in that it was highly localised to, and associated with damaged tissue. Additionally, we found no indication that the mode of preservation had a major impact on melanization: black skin was present in live birds, skins and birds preserved in alcohol (Supplementary Table 11 and 12), and was not related to the age of the specimen (*p*-value > 0.61 in all models; Supplementary Tables 5–7).

### Black skin is labile both in time and species

Preliminary results show that, as previously suggested, black skin is even more widespread in bird chicks^7^ and is lost ontogenetically (Supplementary Fig. 1). This is consistent with black skin as a potential UV protector, as naked or partially feathered chicks are even more vulnerable to UV. Anecdotal evidence also suggests that some birds, e.g., blue jay (*Cyanocitta cristata*), Eurasian magpie (*Pica pica*) and red cardinal (*Cardinalis cardinalis*), have black skin when they lose feathers during molt (Supplementary Fig. 4). However, examination of museum specimens of these species and live specimens of *Cyanocitta cristata* (observed in September 2018) showed no black skin, indicating that this might be a seasonal trait associated with loss of feathers (consistent with our results), or that individuals vary in skin colour. We cannot exclude the latter as we found multiple species (±20%) that showed some variation in skin colour (Supplementary Table 10; Supplementary Fig. 3). This also indicates that the number of species represented here form a lower boundary, and more species are expected to have black skin.

## Conclusions

Our results indicate that black skin evolved over 100 times, across the bird phylogeny. While, as often is the case, unknown trade-offs influence this evolution, a strong association with exposure to UV radiance is present. As a result, black skin tends to be more common towards the equator, an ecogeographic rule known as Gloger’s rule.

Even though we have sampled an extensive part of bird diversity, our results provide but a starting point for future research. One such opportunity is the use of reflectance data (its absence is a limitation of this study) for both feather and skin colour, to identify any potential fine-scale patterns. On larger scales, taking black skin into account could give further insight into the ecological relevance of coloured integument in an ontological, ecological, and evolutionary framework. There is ample variation on a spatial level and a temporal scale, both within species and perhaps even within individuals. As such, this study investigates an extra dimension to bird colouration and should stimulate considerable future research, both descriptive and experimental.

## Methods

### Specimen collection and skin colour assessment

We examined 3610 skins of 2259 species of birds (almost 1 in 4 bird species) spanning all but 7 genera and all families (taxonomy following Clements^42^). We also looked at an additional 18 extinct genera. We looked at skin on two locations on the body: (1) ventrally and (2) on the back of the head, near the neck region. To do so we lifted, or moved away, feathers and registered whether the skin was black, yellow or red. Additionally, we looked at skin from the neck, dorsally and under the wings in a pilot study of 75 species but did not find any black skin, even when black skin was present on the head. We coded skin as either black or non-black. Non-black skin was either yellow or red, but examination of recently deceased birds showed that the yellow skin is probably the result of colour loss associated with pigments (e.g., carotenoids) and blood flow. We collected data from adult specimens when possible. For six species we only had juvenile specimens available. For nine species life stage was unavailable. Neither juveniles nor specimens with unknown life stage had black skin (Supplementary Data 1). When available, we looked at one male and one female specimen. When specimens were scored as black we looked at all specimens available, with a maximum of five specimens per sex. Specimens originated from the natural history collection of the Royal Belgian Institute for Natural Sciences (RBINS, permission granted by Olivier Pauwels), the Royal Museum for Central Africa (RMCA, permission granted by Alain Reygel), the Museum of Comparative Zoology (MCZ, permission granted by Jeremiah Trimble), the American Natural History Museum (ANHM, permission granted by Bentley Bird) and the Natural History Museum (NHM). Additionally, we examined 47 specimens in the alcohol collections of the RMCA and 15 live or recently deceased specimens at Wellfleet Bay Wildlife Sanctuary, Joppa Flats, Manomet, the TEREC collection at UGent (Supplementary Table 12). All data was collected by one person (M.P.J.N) with the help of two other persons (S.P. and R.C). Colour data for *Eutrichomyas rowleyi* and *Xenoperdus obscuratus* were by provided by Martin Päckert (Naturmuseum Senckenberg) and Jon Fjeldså (Natural History Museum of Denmark).

### Choice of phylogenetic tree

All phylogenetic analyses were run on the complete Bayesian maximum clade credibility species-level avian phylogeny from the Bird Tree Project^43^, built based on both genetic and taxonomic information and the higher-order relationship backbone from Hackett et al.^44^. If specific species were not present in the tree due to recent taxonomical changes we coded it as a closely related congeneric species. To test for phylogenetic robustness we also ran a second set of analyses using a MCC genetic-only tree^43^.

### Ancestral state estimation

We used the function “ace” of the ape package^45^ to determine whether an all rates different (ARD) model was significantly better than an all rates equal (ER) model. We then used the best model (ARD) model to simulate 100 unique ancestral state estimations using an MCMC approach to sample character histories from their posterior probability distribution in a process known as stochastic mapping. The function “densityMap” in the package phytools^46,47^ was used to map the posterior density of the trait by plotting each possible character history in proportion to its probability. Traits were categorised as either having black skin or not. This approach included specimens in which at least one specimen was observed with black skin.

### (Phylogenetic) comparative analyses

Phylogenetic signal strength of black skin was measured using Fritz and Purvis’ *D*-test for binary variables^48^, as implemented in the function “phylo.d” in the CAPER package^49^. Since phylogenetic comparative analyses do not allow for repeated measures we divided the data in four subsets: the neck with male priority in case of duplication, the neck with female priority in case of duplication, ventral with male priority in case of duplication and ventral with female priority in case of duplication. However, since black skin evolved ventrally in only 11 genera, ventral analyses were excluded.

We used phylogenetic comparative methods to test whether black skin is associated with a set of life history traits. We controlled for phylogenetic non-independence using a phylogenetic logistic regression implemented in the R package phylolm^50^. *R*² values were calculated using the rr2 package^51^. Physical traits tested were colour of feather overlying the observed skin, presence/absence of sexual dichromatism and maximum known mass. The colours of feathers overlying the skin were assessed on the actual specimen as either white, black, grey, brown, green, yellow, blue, orange, purple, or pink. To exclude colour definition ambiguity we ran an extra analysis with feather colour coded as defined by handbook of the birds of the world alive (HBW)^52^ (see Supplementary Note 1 and Supplementary Table 13). Apart from singular, dominant colour we added the category barred if a barred pattern without dominant colour was present. The handbook of the birds of the world alive (HBW)^52^ was used to score sexual dichromatism as either present or absent based on the description and the associated plates. Sexually dichromatism included were differences in skin colour and plumage, while differences in eye-colour were excluded. HBW was also used to quantify maximal known mass. If the mass was not known for a particular species, we used the mass of a similarly sized congeneric species. Climatological variables tested were latitude of mean breeding range (absolute, in order to test for association with equatorial regions)^53^, annual mean UV-B radiation (jm^−2^/day) at coordinates of breeding range centroid (in M jm^2^/day)^54^, average precipitation at breeding range (mm/day) and the maximum temperature (°C) at coordinates of mean breeding range, obtained from the NASA Langley Research Centre (LaRC) POWER Project. For temperature the upper limit was used, as upper critical thermal limits drive thermal adaptations^28^. Finally, we also used the presence/absence of high interaction species (defined as species that are both obligatory and facultative colonial species, as well as species that form groups, share roosts  or are communal or social hole breeders) obtained from HBW, and whether birds occupied a covered habitat, which we defined as being strictly confined to forest and/or shrub habitat, as defined by the IUCN redlist website^55^. Continuous data was standardised and mass and UV radiance were log-transformed.

### Niche modelling

Maxent^56^ a maximum entropy modelling tool commonly used to model species distribution, was used to investigate the response of black skin in function of four different climatic variables. In this analysis we clumped all species with black skin into one hypothetical black skinned species. For each species with black skin, occurrence data was downloaded from The Global Biodiversity Information Facility (gbif)^57^. To avoid sampling bias (i.e., widespread species that are more commonly observed at locations with lots of birdwatchers, e.g., the UK) each occurrence was assigned to a 2 × 2 degree grid. For each species we then sampled 50 (or less when not available) random occurrences out of these 2 × 2 degree grids. This resulted in 5128 coordinates that were used in the distribution modelling. Variables used were Annual mean UV-B radiation (jm^−2^ day^−1^), average minimum temperature (°C), average maximum temperature (°C) and average precipitation (mm). UV radiance was collected from gIUV^54^. All other variables were yearly averaged over a span of 30 years and were downloaded from Worldclim 2^58^. The main output consists of a predicted distribution of a species (or a trait in this case). This predicted distribution model was compared with a bird diversity map^59^ that was normalised using the maximal diversity value to identify black skin hotspots and coldspots. While the Maxent model is being trained it keeps track of how much each environmental predictor contributes to the predicted trait distribution. This is converted to percentages at the end of the training process which results in the variable contribution table (Supplementary Fig. 6)^60^. Additionally, Maxent produces response curves for each variable (Supplementary Fig. 6). These graphs show the predicted probability of suitable conditions on the *y*-axis and the values of the predictor variable on the *x*-axis^60^. Maps were made using the R package maps^61^.

### TEM and light microscopy

To verify that presence and/or greater abundance of melanin explains black skin colour, we used optical and transmission electron microscopy on skin samples of one individual each for *Morus bassanus* (black) and *Garrulus glandarius* (red). Samples of black and yellow/pink skin were embedded in Epon (Electron Microscopy Solutions, Hatfield, PA, USA) following standard protocol. We stained thin (100 nm) sections in Uranyless/lead citrate and examined them on a JEOL JEM 1010 (Jeol, Ltd, Tokyo, Japan) transmission electron microscope.

### Reporting summary

Further information on research design is available in the Nature Research Reporting Summary linked to this article.

## Supplementary information

Supplementary Information

Reporting Summary

Description of Additional Supplementary Files

Supplementary Data 1

## Data Availability

All data used in this study are available in Supplementary Data 1.
