# Supplementary material for: Exposure to UV radiance predicts repeated evolution of concealed black skin in birds
Source: Nat Commun. 2020 May 15;11:2414. doi: 10.1038/s41467-020-15894-6 (PMC7229023; doi:10.1038/s41467-020-15894-6)
Supplement: Supplementary file 1 — Supplementary Information [file 41467_2020_15894_MOESM1_ESM.pdf]

Supplementary Information

Exposure to UV radiance predicts repeated evolution of concealed black skin in birds

Nicolaï et al.

Table of contents

Supplementary Note 1: Colour data collection

Supplementary Figures 1-6

Supplementary Tables 1-13

Supplementary Note 1: Colour data collection

Even though all colour data was collected by one person, assigning colours to categories, with or without techniques like spectrophotometry or photography, becomes subjective at one point. As such we performed a sensitivity analysis by collecting feather colour data from descriptions in HBW. In case of doubt, e.g. when two colours where mentioned or when a colour was considered “variable”, we gave ourselves the benefit of the doubt by assigning the colour that both HBW and we identified. Sometimes descriptions were unambiguous. As such terms such as “rusty”, “sandy”, “cinnamon”, “clay”, “tan”, “buff”, “chestnut”, “rusty”, “dusky”, “rufous” and “russet” were assigned brown; terms such as “silver”, “sooty”, “mousy brown”, “clouded”, “isabelline”, and “ashy” were used for grey; terms such as “golden”, “fulvous” and “pale buff” where considered yellow; terms such as “darker than liver brown”, “dark chocolate brown”, and “darker than grey” were considered black; terms such as “creamy” and “pale grey” where considered white. Terms such as olive were considered more variable and could either be green, yellow or brown. More specifically we found that for olive the colour was context dependent. For example, colours defined as (dark) olive (green) were consistently seen as brown (e.g. in *Calyptocichla serinus*, *Philepitta castanea*, *Seiurus aurocapilla*,*Sericornis arfakianus*, *Chlorocharis emiliae*, *Rhynchocyclus olivaceus*, *Telespiza cantans*, *Meliphaga lewinii*, *Icteria virens*, *Deltarhynchus flammulatus*, *Chlorocichla falkensteini* (as *phyllastrephus*), *Anthipes monileger*, *Antilophia galeata*). On the other hand, bright, dull or paler than olive was often seen as yellow (*Atalotriccus pilaris*, *Baeopogon indicator*, *Cyanoliseus patagonus*, *Dacnis lineata*, *Eulacestoma nigropectus*, *Melichneutes robustus*, *Myiornis auricularis*, *Tachyphonus surinamus*, *Ara militaris*). Finally, olive was also assigned as green (e.g. in *Aethopyga shelleyi* and *Zimmerius viridiflavus*).

The results of this sensitivity analysis indicate that overall 85% of the colour assignments correspond between different methods (supplementary table 13). Furthermore, all birds with black skin were assigned correct colours and white feathers on the neck (for which statistical analyses were done) were almost always identified correctly (13 mismatches). Most of the mismatches were between dark colours (brown, grey, black) and furthermore, many of these mismatches are symmetrical (e.g. grey <-> black is 13 vs black<-> grey is 20). Given the high hit-rate, and the nature of the mismatches, we considered our results robust to subjective colour identification. Overall trends present in correlative analyses gave similar results showing that observed patterns are robust (Supplementary tables 6-8).

Supplementary figures

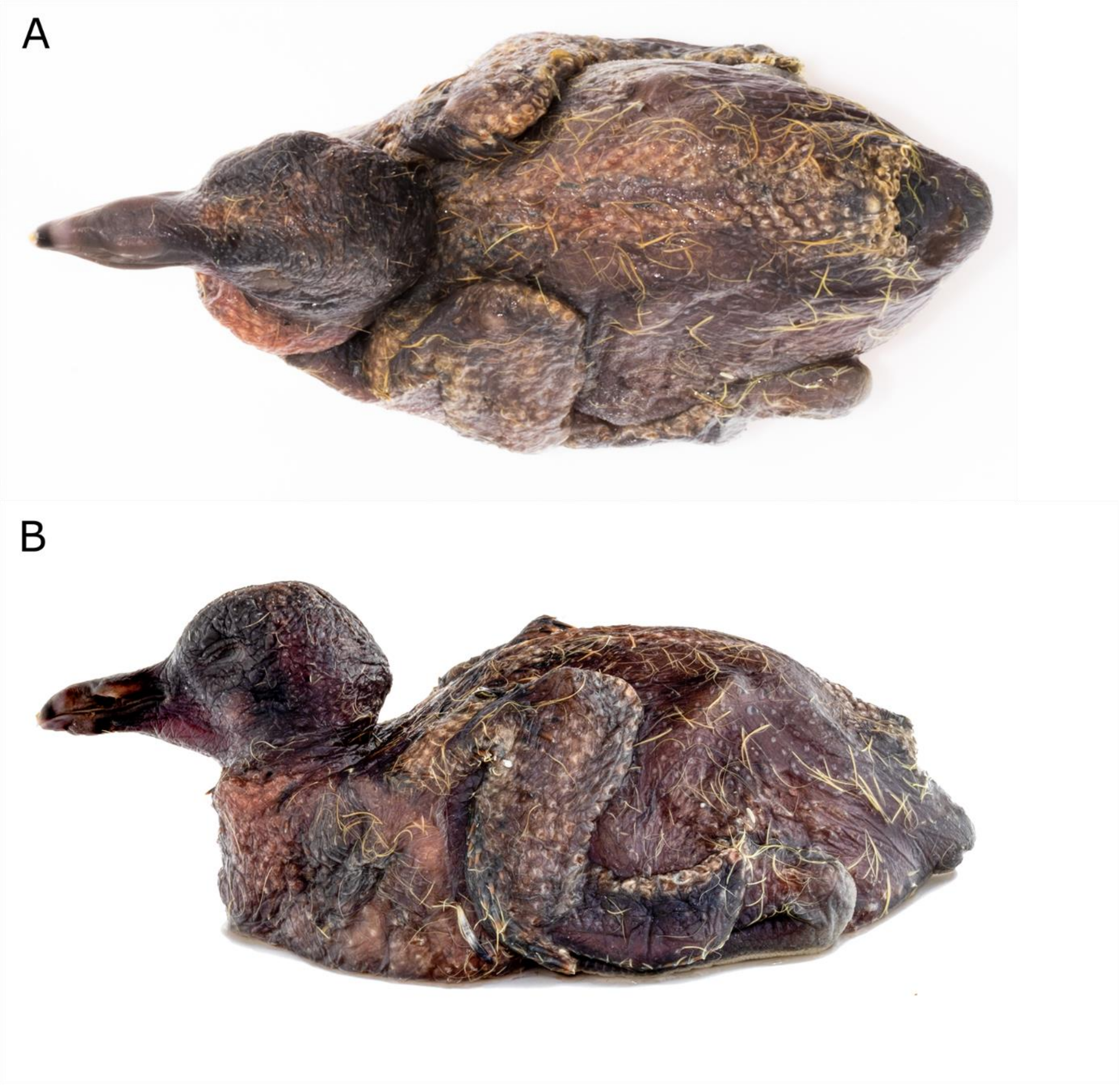

Supplementary Figure 1. Photo of a nestling of the Eurasian collared dove (*Streptopelia decaocto*) showing a highly melanized head, medium melanized dorsum and red throat and flanks.

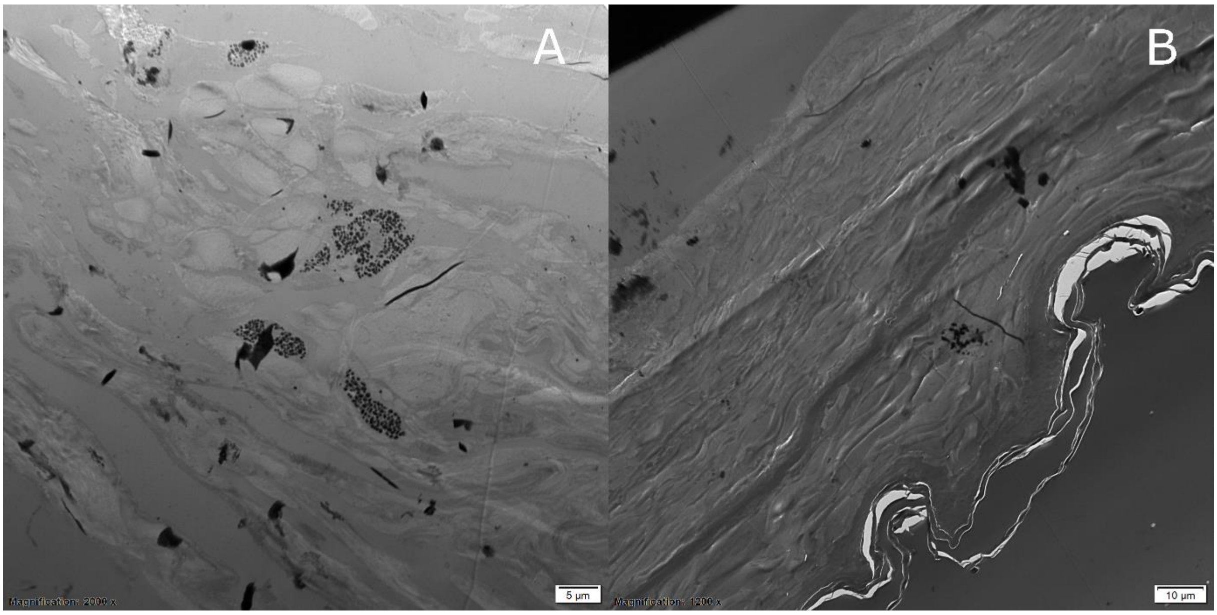

Supplementary Figure 2. TEM for (A) *Morus bassanus* (black skin from one specimen) (scale bar 5µm, white arrows mark melanosomes) and (B) for *Garrulus glandarius* (red skin from one specimen) (scale bar 10 µm) where melanosomes were absent (although low levels of melanin are observed scattered throughout the skin; see figure 1 in main text).

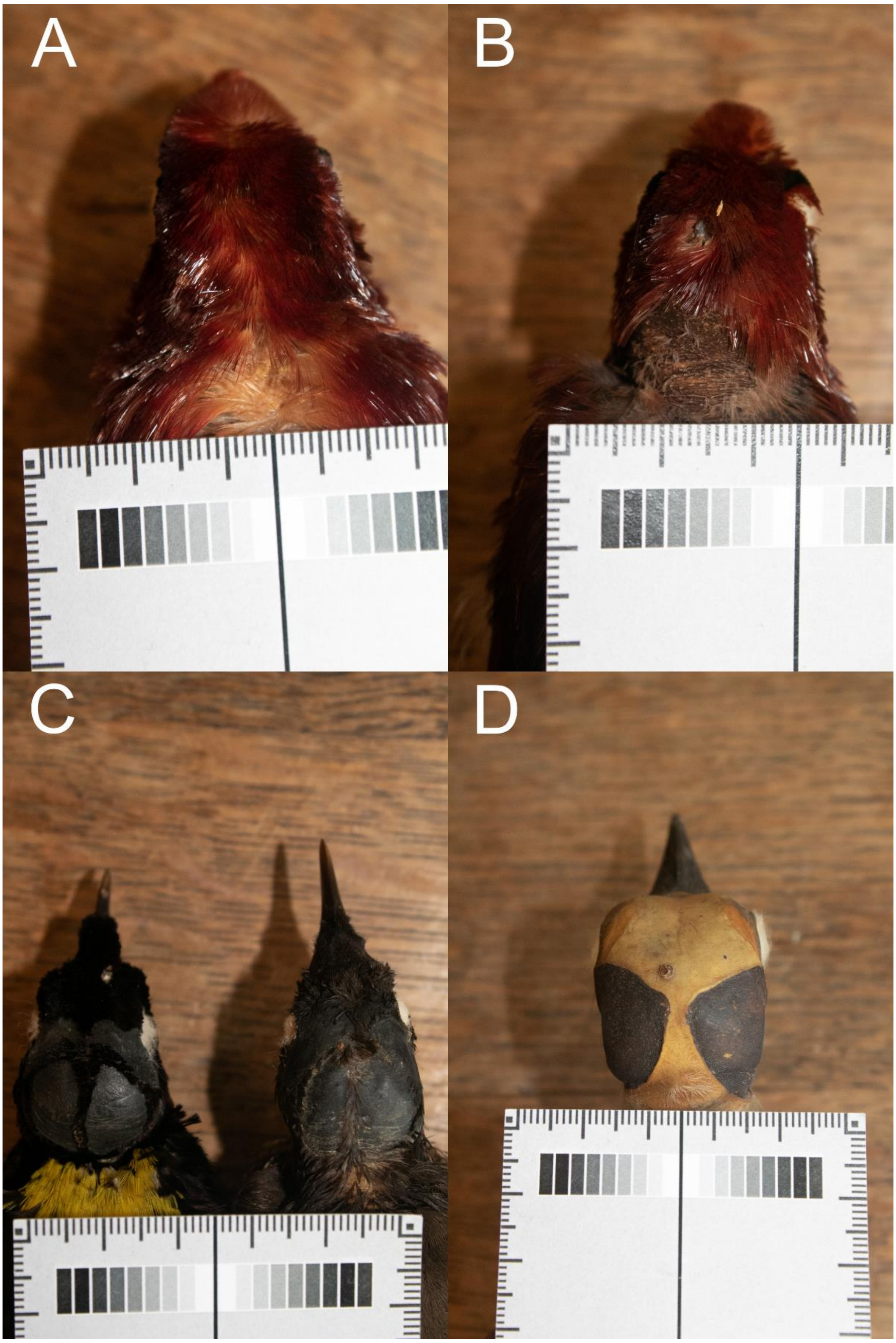

Supplementary Figure 3. (A-B) Presence of sexual dichromatism in skin colour of the King bird-of-paradise (*Cicinnurus regius*). (C) Absence of sexual dichromatism in skin colour of Wilsons's bird-of-paradise (*Cicinnurus respublica*). (D) Presence of dichromatism within one individual of White-necked rockfowl (*Picathartes gymnocephalus*).

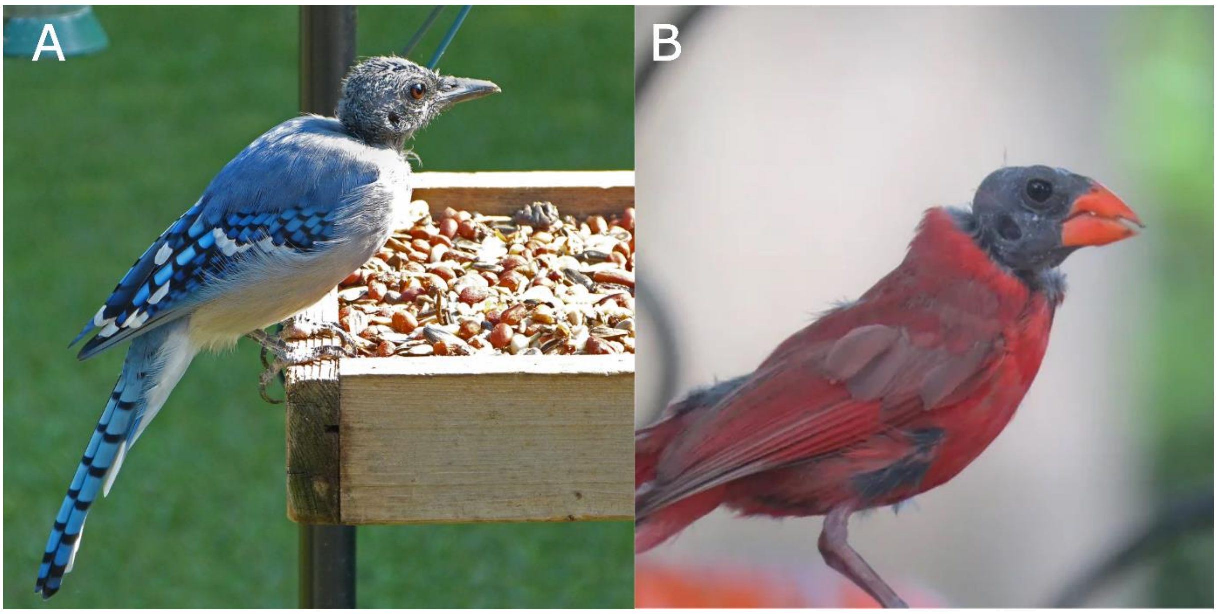

Supplementary Figure 4. Photo showing (A) a bald blue jay (*Cyanocitta cristata*) and (B) red cardinal (*Cardinalis cardinalis*) with black skin. Image (A) from Project FeederWatch at the Cornell Lab of Ornithology and submitted by Bob Vuxinic. Image (B) from Reddit and submitted by user fruitysteve.

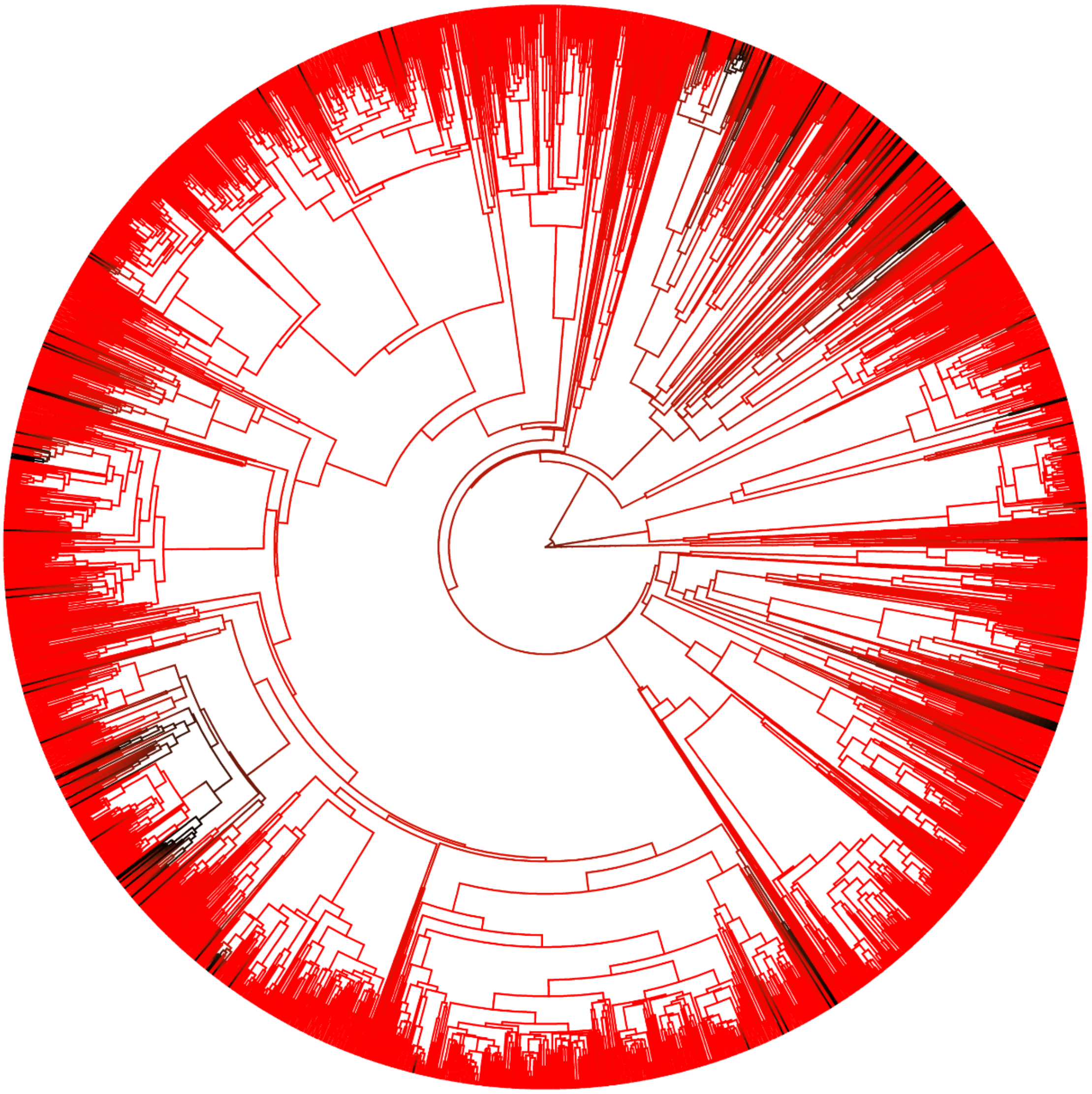

Supplementary Figure 5. Ancestral state estimation for black skin of the heads of male birds. Branches of the phylogenetic tree are black when the reconstructed skin colour was black, and red when this was non-black.

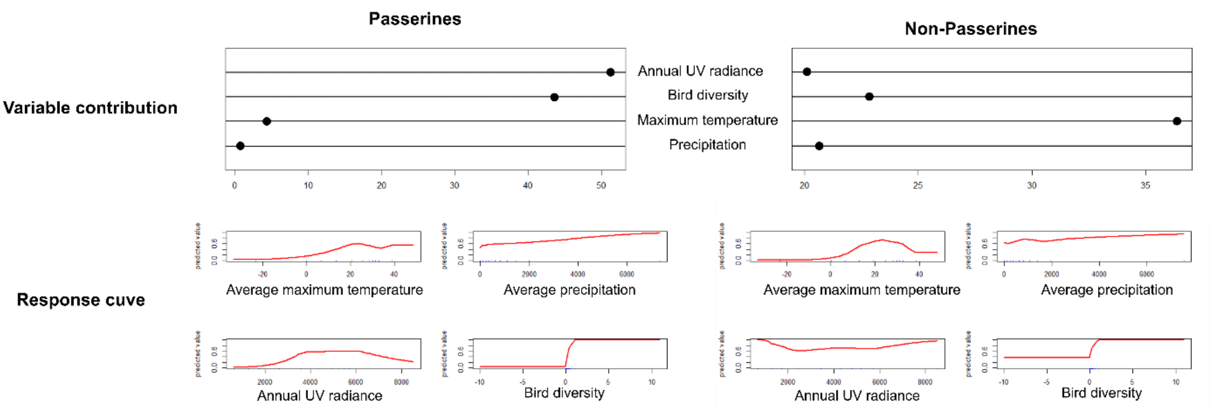

Supplementary Figure 6. Variable contribution (upper pane) and response curve of four variables tested (Average maximum temperature, Average precipitation, Annual UV radiance and Bird diversity) for passerines (left) and non-passerines (right).

Supplementary tables

Supplementary Table 1. Model comparison for the phylogenetically controlled logistic regressions used to explore five working hypotheses for the function of black skin. These hypotheses are: 1) Gloger's rule (which predicts more black skin at low latitudes; 2) UV protection 1(which predicts black skin in birds that are more exposed to UV radiance and have less protection because of the absence or only lightly coloured feathers); 3) UV protection 2 (which predicts, in addition to the factors of UV protection 1, that open habitats are expected to be more associated with black skin compared to closed habitats as a result of exposure to UV radiance); 4) Bacterial protection hypothesis (which predicts that lightly coloured feathers, high temperatures, high moisture levels and a colonial lifestyle are more prone to have black skin as a form of bacterial protection) and 5) Thermoregulation (which predicts more black skin in birds living in cold and dry areas). The likelihood (LnL), Akaike Information Criteria (AIC) and weighed AIC for all models tested are shown, both for male and female data and for three different datasets: all birds combined, non-passerines only, and passerines only for: complete phylogentic tree | the molecular only phylogenetic tree.

**All birds**	**Male (*n*=2109)**				**Female (*n*=2109)**			
	LnL	AIC	AIC weight	R^2	LnL	AIC	AIC weight	R^2
latitude (Gloger's rule)	-446.6 | -365.6	899.2 | 737.2	0.00 | 0.00	0.07 | 0.06	-440.9 | 363.8	887.8 | 733.5	0.00 | 0.00	0.09 | 0.06
feather + UV radiance (UV protection 1)	-346.9 | -285.5	723.8 | 601.0	0.82 | 0.67	0.31 | 0.30	-367.8 | -305.5	765.7 | 641.1	0.51 | 0.73	0.27 | 0.24
feather + UV radiance+closed habitat (UV protection 2)	-348.3 | -286.1	728.7 | 604.2	0.07 | 0.14	0.31 | 0.30	-368.20 | -305.5	768.40 | 643.1	0.13 | 0.27	0.26 | 0.24
feather + water + colonial + max temperature + total precipitation (Bacterial protection)	-346.9 | -284.8	727.8 | 603.5	0.11 | 0.19	0.31 | 0.30	-366.20 | -308.8	766.40 | 651.6	0.36 | 0.00	0.27 | 0.23

max temperature + total precipitation (thermoregulation)	-444.6 | -365.4	897.3 | 738.7	0.00 | 0.00	0.08 | 0.06	-438.90 | -363.4	885.80 | 734.8	0.00 | 0.00	0.09 | 0.06
**Non-passerines**	**Male (*n*=934)**				**Female (*n*=934)**			
	LnL	AIC	AIC weight		LnL	AIC	AIC weight	
latitude (Gloger's rule)	-221.0 | -190.6	447.9 | 387.1	0.00 | 0.00	0.05 | 0.04	-220.9 | -190.1	447.8 | 386.2	0.00 | 0.00	0.05 | 0.05
feather + UV radiance (UV protection 1)	-151.5 | -139.5	333.0 | 308.9	0.03 | 0.00	0.40 | 0.35	-171.7 | -153.0	373.3 | 336.0	0.00 | 0.00	0.30 | 0.27
feather + UV radiance+closed habitat (UV protection 2)	-152.5 | -136.1	337.0 | 304.1	0.00 | 0.00	0.40 | 0.37	-161.8 | -143.9	355.5 | 319.7	0.09 | 0.00	0.35 | 0.32
feather + water + colonial + max temperature + total precipitation (Bacterial protection)	-145.8 | -126.7	325.7 | 287.4	0.97 | 1.00	0.43 | 0.42	-158.4 | 308.2	350.8 | 308.2	0.91 | 1.00	0.37 | 0.36
max temperature + total precipitation (thermoregulation)	-216.0 | -185.7	440.0 | 379.3	0.00 | 0.00	0.08 | 0.07	-216.5 | -185.8	441.0 | 379.5	0.00 | 0.00	0.08 | 0.07
**Passerines**	**Male (*n*=1175)**				**Female (*n*=1175)**			
	LnL	AIC	AIC weight		LnL	AIC	AIC weight	
latitude (Gloger's rule)	-222.10 | -170.9	450.10 | 347.9	0.00 | 0.00	0.10 | 0.08	-218.80 | -171.9	443.60 | 349.9	0.00 | 0.11	0.12 | 0.07
feather + UV radiance (UV protection 1)	-194.30 | -151.5	418.50 | 333.0	0.61 | 0.59	0.24 | 0.20	-201.20 | -159.4	430.40 | 346.8	0.55 | 0.52	0.20 | 0.15
feather + UV radiance+closed habitat (UV protection 2)	-193.80 | -150.9	419.60 | 333.7	0.35 | 0.41	0.24 | 0.20	-200.50 | -158.8	431.00 | 347.6	0.41 | 0.35	0.20 | 0.15
feather + colonial + max temperature + total precipitation (Bacterial protection)	-195.20 | -157.2	424.4 | 348.4	0.03 | 0.00	0.23 | 0.16	-201.70 | -165.3	435.30 | 362.5	0.05 | 0.00	0.20 | 0.11
max temperature + total precipitation (thermoregulation)	-223.2 | -172.1	454.4 | 352.2	0.00 | 0.00	0.10 | 0.07	-219.8 | -173.1	447.6 | 354.2	0.00 | 0.00	0.11 | 0.06

Supplementary Table 2. The results of phylogenetically controlled logistic regressions used to explore five working hypotheses for the function of black skin. These hypotheses are: 1) Gloger's rule (which predicts more black skin at low latitudes; 2) UV protection 1(which predicts black skin in birds that are more exposed to UV radiance and have less protection because of the absence or only lightly coloured feathers); 3) UV protection 2 (which predicts, in addition to the factors of UV protection 1, that open habitats are expected to be more associated with black skin compared to closed habitats as a result of exposure to UV radiance); 4) Bacterial protection hypothesis (which predicts that lightly coloured feathers, high temperatures, high moisture levels and a colonial lifestyle are more prone to have black skin as a form of bacterial protection) and 5) Thermoregulation (which predicts more black skin in birds living in cold and dry areas). Statistics (variable estimates and standard errors, as well as *Z*-value and corresponding p-value for two-sided tests without multiple comparison adjustments) are shown for male and female datasets of all birds for both the complete and the molecular only phylogenetic tree.

**Model**	**Factor**	**Estimate**	**SE**	***Z*-value**	***p*-value**

**latitude (Gloger's Rule) male | female, complete tree || molecular tree only**					
	Intercept	-2.42 | -2.45 ||-2.40 | -2.39	0.18 | 0.19 || 0.20 | 0.20	-13.13 | -12.89 || -11.95 | -11.74	0.00 | 0.00 || 0.00 | 0.00
	Latitude	-1.28 | -1.17 || -1.37 | -1.43	0.53 | 0.52 || 0.57 | 0.57	-2.42 | -2.24||-2.41 | -2.52	0.02 | 0.02 || 0.02 | 0.01
**feather + UV radiance (UV protection 1) male | female**					
	Intercept	-10.05 | -8.24 || -10.44 | -9.91	4.64 | 4.22 || 4.63 | 4.36	-2.17 | -1.95 || -2.26 | -2.27	0.03 | 0.00 || 0.02 | 0.02
	White	1.70 | 1.58 || 1.87 | 1.77	0.52 | 0.60 || 0.59 | 0.68	3.31 | 2.63 || 3.15 | 2.60	0.00 | 0.01 || 0.00 | 0.00
	Grey	-0.66 | -0.26 || -0.28 | 0.17	0.51 | 0.56 || 0.55 | 0.62	-1.30 | -0.47 || -0.51 | 0.28	0.19 | 0.64 || 0.61 | 0.78
	Brown	-1.11 | -0.97 || -0.75 | -0.68	0.49 | 0.55 || 0.53 | 0.61	-2.27 | -1.75 || -1.40 | -1.12	0.02 | 0.08 || 0.16 | 0.26
	Green	-1.77 | -2.99 || -1.44 | -2.15	0.68 | 1.03 || 0.73 | 0.93	-2.59 | -2.89 || -1.99 | -2.32	0.01 | 0.00 || 0.05 | 0.02
	Yellow	-0.62 | -0.56 || -0.34 | -0.46	0.56 | 0.63 || 0.59 | 0.68	-1.11 | -0.89 || -0.58 | -0.69	0.27 | 0.37 || 0.56 | 0.49
	Blue	-1.14 | -0.98 || -0.69 | -0.16	0.69 | 0.77 || 0.71 | 0.74	-1.64 | -1.27 || -0.97 | -0.21	0.1 | 0.20 || 0.33 | 0.83
	Orange	-15.22 | -0.56 || -0.30 | -0.21	1639.42 | 1.41 || 0.25 | 1.58	-0.01 | -0.36 || -0.24 | -0.13	0.99 | 0.72 || 0.81 | 0.89
	Purple	-15.25 | -0.98 || -0.42 | -0.30	1437.73 | 1.60 || 1.09 | 1.57	-0.01 | -0.25 || -0.39 | -0.19	0.99 | 0.81 || 0.70 | 0.85
	Black	-1.80 | -1.05 || -1.58 | -0.93	0.55 | 0.58 || 0.60 | 0.64	-3.27 | -1.80 || -2.63 | -1.44	0.00 | 0.07 || 0.00 | 0.15
	Pink	-15.27 | -0.35 || -0.36 | 0.19	2592.00 | 2.14 || 1.99 | 2.17	-0.01 | -0.16 || -0.18 | 0.09	1.00 | 0.87 || 0.86 | 0.93
	Barred	-15.30 | -0.75 || -0.42 | -0.40	1560.85 | 1.20 || 1.17 | 1.23	-0.01 | -0.66 || -0.36 | -0.32	0.99 | 0.51 || 0.72 | 0.75
	Bald	2.39 | 2.29 || 2.45 | 2.28	0.57 | 0.64 || 0.65 | 0.73	4.19 | 3.57 || 3.80 | 3.14	0.00 | 0.00 || 0.00 | 0.00
	UV radiance	8.62 | 6.65 || 8.80 | 8.22	4.87 | 4.43 || 4.85 | 4.55	1.77 | 1.50 || 1.82 | 1.81	0.08 | 0.13 || 0.07 | 0.07
**feather + UV radiance+closed habitat (UV protection 2) male | female, complete tree || molecular tree only**					
	Intercept	-8.95 | -7.90 || -10.37| -10.21	4.31 | 4.28 || 4.48| 4.65	-2.08 | -1.85 || -2.32| -2.20	0.04 | 0.06 || 0.02| 0.03
	White	1.61 | 1.87 || 1.67| 1.61	0.50 | 0.66 || 0.57| 0.67	3.19 | 2.84 || 2.98| 2.42	0.00 | 0.00 || 0.00| 0.02
	Grey	-0.67 | -0.02 || -0.38| -0.03	0.49 | 0.62 || 0.52| 0.63	-1.37 | -0.04 || -0.74| -0.05	0.17 | 0.97 || 0.46| 0.96
	Brown	-1.05 | -0.72 || -0.84| -0.93	0.47 | 0.60 || 0.51| 0.62	-2.25 | -1.20 || -1.66| -1.51	0.02 | 0.23 || 0.10| 0.13
	Green	-1.74 | -2.33 || -1.71| -2.15	0.64 | 0.95 || 0.72| 0.91	-2.71 | -2.46 || -2.37| -2.35	0.01 | 0.01 || 0.02| 0.02
	Yellow	-0.67 | -0.30 || -0.44| -0.99	0.54 | 0.67 || 0.56| 0.75	-1.25 | -0.45 || -0.78| -1.31	0.21 | 0.65 || 0.43| 0.19
	Blue	-1.23 | -0.55 || -0.56| -0.69	0.67 | 0.78 || 0.63| 0.83	-1.83 | -0.70 || -0.89| -0.84	0.07 | 0.48 || 0.38| 0.40
	Orange	-0.62 | 0.05 || -0.42| -0.11	1.07 | 1.37 || 1.19| 1.59	-0.58 | -0.04 || -0.35| -0.07	0.56 | 0.97 || 0.73| 0.95
	Purple	-0.73 | 0.12 || -0.55| -0.28	1.00 | 1.57 || 1.04| 1.60	-0.73 | 0.08 || -0.53| -0.18	0.46 | 0.94 || 0.60| 0.86
	Black	-1.78 | -0.78 || -1.72| -1.12	0.53 | 0.63 || 0.58| 0.66	-3.39 | -1.24 || -2.98| -1.70	0.00 | 0.21 || 0.00| 0.09
	Pink	-0.51 | 0.49 || -0.24| 0.03	1.74 | 1.76 || 1.79| 2.20	-0.29 | 0.28 || -0.13| 0.02	0.77 | 0.78 || 0.89| 0.99
	Barred	-0.75 | -0.31 || -0.52| -0.43	0.96 | 1.13 || 1.10| 1.26	-0.79 | -0.27 || -0.47| -0.34	0.43 | 0.79 || 0.64| 0.73
	Bald	2.27 | 2.60 || 2.26| 2.17	0.55 | 0.70 || 0.62| 0.71	4.08 | 3.73 || 3.65| 3.06	0.00 | 0.00 || 0.00| 0.00
	UV radiance	7.53 | 5.97 || 8.93| 8.68	1.67 | 4.48 || 4.68| 4.88	1.67 | 1.33 || 1.90| 1.78	0.10 | 0.18 || 0.06| 0.08
	Covered habitat	0.06 | 0.01 || 0.00| -0.09	0.34 | 0.19 || 0.19| 0.21	0.34 | 0.04 || -0.03| -0.46	0.74 | 0.97 || 0.97| 0.65
**feather + colonial + max temperature + total precipitation (Bacterial protection) male | female, complete tree || molecular tree only**					
	Intercept	-1.72 | -1.07 || -1.95 | -0.08	0.55 | 0.51 || 0.63 | 1.23	-3.14 | -2.11 || -3.09 | -0.06	0.00 | 0.03 || 0.00 | 0.95
	White	1.30 | 0.59 || 1.57 | 0.36	0.49 | 0.49 || 0.57 | 0.37	2.64 | 1.22 || 2.73 | 0.97	0.01 | 0.22 || 0.00 | 0.33
	Grey	-0.63 | -0.93 || -0.33 | -0.27	0.46 | 0.47 || 0.51 | 0.32	-1.35 | -1.98 || -0.65 | -0.83	0.18 | 0.05 || 0.51 | 0.41
	Brown	-1.13 | -1.70 || -0.86 | -0.36	0.47 | 0.49 || 0.51 | 0.37	-2.41 | -3.46 || -1.70 | -0.98	0.02 | 0.00 || 0.09 | 0.33
	Green	-2.02 | -2.48 || -1.28 | -1.64	0.68 | 0.68 || 0.66 | 0.33	-2.97 | -3.64 || -1.94 | -4.97	0.00 | 0.00 || 0.05 | 0.00
	Yellow	-0.54 | -1.08 || -0.37 | -0.95	0.50 | 0.54 || 0.55 | 0.32	-1.09 | -2.00 || -0.68 | -3.01	0.27 | 0.04 || 0.49 | 0.00
	Blue	-1.72 | -1.87 || -0.83 | -0.35	0.76 | 0.76 || 0.68 | 0.38	-2.26 | -2.47 || -1.22 | -0.91	0.02 |0.01 || 0.22 | 0.36
	Orange	-0.65 | -12.18 || -0.49 |-10.67	1.02 | 334.41 || 1.22 | 107.62	-0.64 | -0.04 || -0.40 | -0.10	0.52 | 0.97 || 0.69 | 0.92
	Purple	-0.73 | -12.47 || -0.62 | -10.88	0.94 | 424.33 || 1.10 | 118.60	-0.77 | -0.03 || -0.57 | -0.09	0.44 | 0.98 || 0.57 | 0.93
	Black	-1.86 | -1.45 || -1.73 | -0.41	0.52 | 0.50 || 0.57 | 0.40	-3.57 | -2.88 || -3.03 | -1.03	0.00 | 0.00 || 0.00 | 0.30
	Pink	-0.77 | -12.81 || -0.56 | -11.25	1.64 | 484.34 || 1.85 | 195.55	-0.47 | -0.03 || -0.30 | -0.06	0.64 | 0.98 || 0.76 | 0.95
	Barred	-0.58 | -12.15 || -0.44 | -10.67	0.84 | 254.10 || 1.11 | 76.22	-0.68 | -0.05 || -0.40 | -0.14	0.50 | 0.96 || 0.69 | 0.89
	Bald	2.09 | 1.47 || 2.27 | 0.87	0.54 | 0.54 || 0.63 | 0.55	3.84 | 2.74 || 3.62 | 1.58	0.00 | 0.01 || 0.00 | 0.11
	Colonial	0.34 | 0.42 || 0.15 | 0.04	0.22 | 0.21 || 0.24 | 0.08	1.52 | 2.03 || 0.63 | 0.48	0.13 | 0.04 || 0.53 | 0.63
	Max temperature	0.36 | 0.34 || 0.41 | 0.09	0.38 | 0.36 || 0.40 | 0.12	0.94 | 0.95 || 1.04 | 0.73	0.35 | 0.34 || 0.30 | 0.47
	Total precipitation	-0.79 | -1.17 || -0.82 | -0.16	0.54 | 0.51 || 0.52 | 0.17	-1.47 | -2.26 || -1.59 | -0.94	0.14 | 0.02 || 0.11 | 0.35
**max temperature + total precipitation (Thermoregulation) male | female, complete tree || molecular tree only**					
	Intercept	-3.15 | -3.05 || -3.33 | -3.35	0.39 | 0.38 || 0.43 | 0.43	-8.0576 | -7.94 || -7.79 | -7.75	0.00 | 0.00 || 0.00 | 0.00
	Max	1.12 | 0.97 || 1.15 | 1.25	0.49 | 0.48 || 0.54 | 0.54	2.2572 | 2.02 || 2.14 | 2.31	0.02 | 0.04 || 0.03 | 0.02
	Total precipitation	-1.05 | -1.08 || -0.38 | -0.53	0.56 | 0.56 || 0.53 | 0.53	-1.8698 | -1.92 || -0.72 | 1.00	0.06 | 0.06 || 0.47 | 0.32

Supplementary Table 3. The results of phylogenetically controlled logistic regressions used to explore five working hypotheses for the function of black skin. These models are: These hypotheses are: 1) Gloger's rule (which predicts more black skin at low latitudes; 2) UV protection 1(which predicts black skin in birds that are more exposed to UV radiance and have less protection because of the absence or only lightly coloured feathers); 3) UV protection 2 (which predicts, in addition to the factors of UV protection 1, that open habitats are expected to be more associated with black skin compared to closed habitats as a result of exposure to UV radiance); 4) Bacterial protection hypothesis (which predicts that lightly coloured feathers, high temperatures, high moisture levels and a colonial lifestyle are more prone to have black skin as a form of bacterial protection) and 5) Thermoregulation (which predicts more black skin in birds living in cold and dry areas). Statistics (variable estimates and standard errors, as well as *Z*-value and corresponding p-value for two-sided tests without multiple comparison adjustments) are shown for male and female datasets of all non-passerines for both the complete and the molecular only phylogenetic tree.

**Model**	**Factor**	**Estimate**	**SE**	***Z*-value**	***p*-value**
**latitude (Gloger's Rule) male | female, complete tree || molecular tree only**					
	Intercept	-2.47 | -2.47 || -2.54 | -2.50	0.30 | 0.27 || 0.31 | 0.30	-8.24 | -9.00 || -8.12 | -8.41	0.00 | 0.00 || 0.00 | 0.00
	Latitude	0.77 | 0.59 || 0.99 | 0.81	0.52 | 0.53 || 0.54 | 0.54	1.50 | 1.11 || 1.84 | 1.50	0.13 | 0.27 || 0.07 | 0.13
**feather + UV radiance (UV protection 1) male | female, complete tree || molecular tree only**					
	Intercept	-19.08 | -16.25 || -22.67 | -21.91	994.53 | 18.62 || 296.89 | 28.97	-0.02 | -0.87 || -0.08 | -0.76	0.98 | 0.38 || 0.94 | 0.45
	White	16.66 | 9.21 || 14.39 | 9.78	994.52 | 18.56 || 296.87 | 28.87	0.02 | 0.50 || 0.05 | 0.34	0.99 | 0.62 || 0.96 | 0.73
	Grey	15.78 | 8.31 || 13.19 | 8.25	994.52 | 18.56 || 296.87 | 28.87	0.02 | 0.45 || 0.04 | 0.29	0.99 | 0.65 || 0.96 | 0.78
	Brown	15.63 | 8.40 || 12.71 | 8.53	994.52 | 18.56 || 296.87 | 28.87	0.02 | 0.45 || 0.04 | 0.30	0.99 | 0.65 || 0.97 | 0.77
	Green	1.41 | 8.20 || 3.97 | 7.91	1011.80 | 18.56 || 297.23 |28.87	0.00 | 0.44 || 0.01 | 0.27	1.00 | 0.65 || 0.99 | 0.78
	Yellow	1.41 | 8.39 || 3.99 | 8.19	1165.79 | 18.56 || 299.94 |28.87	0.00 | 0.45 || 0.01 | 0.28	1.00 | 0.65 || 0.99 | 0.78
	Blue	15.70 | 8.47 || 12.82 | 8.34	994.52 | 18.56 || 296.87 | 28.87	0.02 | 0.46 || 0.04 | 0.29	0.99 | 0.65 || 0.96 | 0.78
	Orange	1.41 | 8.43 || 4.00 | 8.37	1842.24 | 18.56 || 334.93 | 28.89	0.00 | 0.45 || 0.01 | 0.29	1.00 | 0.65 || 0.99 | 0.78
	Purple	1.41 | 8.41 || 3.95 | 8.4	1293.80 | 18.56 || 304.73 | 28.87	0.00 | 0.45 || 0.01 | 0.29	1.00 | 0.65 || 0.99 | 0.77
	Black	15.34 | 8.47 || 12.09 | 8.74	994.52 | 18.56 || 296.87 | 28.87	0.02 | 0.46 || 0.04 | 0.30	0.99 | 0.65 || 0.97 | 0.76
	Pink	1.41 | 8.42 || 3.97 | 8.51	1607.88 | 18.57 || 316.29 | 28.88	0.00 | 045 || 0.01 | 0.29	1.00 | 0.65 || 0.99 | 0.77
	Barred	1.40 | 8.32 || 3.91 | 8.17	1334.19 | 18.56 || 304.55 | 28.88	0.00 | 0.45 || 0.01 | 0.28	1.00 | 0.65 || 0.99 | 0.78
	Bald	17.56 | 9.99 || 14.53 | 10.00	994.52 | 18.57 || 296.87 | 28.88	0.02 | 0.54 || 0.05 | 0.35	0.99 | 0.59 || 0.96 | 0.73
	UV radiance	2.42 | 8.02 || 9.10 | 13.33	2.35 | 1.52 || 3.54 | 2.42	1.03 | 5.27 || 2.57 | 5.50	0.30 | 0.00 || 0.01 | 0.00
**feather + UV radiance+closed habitat (UV protection 2) male | female**					
	Intercept	-19.58 | -12.95 || -24.45 | -20.97	1084.76 | 26.50 || 862.05 | 31.69	-0.02 | -0.49 || -0.03 | -0.66	0.99 | 0.63 || 0.98 | 0.51
	White	17.26 | 10.12 || 16.41 | 8.60	1084.76 | 26.47 || 862.05 | 31.57	0.02 | 0.38 || 0.02 | 0.31	0.99 | 0.70 || 0.98 | 0.76
	Grey	15.92 | 9.25 || 15.07 | 9.73	1084.76 | 26.48 || 862.05 | 31.57	0.01 | 0.35 || 0.02 | 0.27	0.99 | 0.73 || 0.99 | 0.79
	Brown	15.90 | 9.30 || 14.99 | 8.61	1084.76 | 26.48 || 862.05 | 31.57	0.01 | 0.35 || 0.02 | 0.26	0.99 | 0.73 || 0.99 | 0.79
	Green	1.14 | 9.04 || 2.07 | 8.29	1110.15 | 26.47 || 869.19 | 31.57	0.00 | 0.34 || 0.00 | 0.26	1.00 | 0.73 || 1.00 | 0.79
	Yellow	1.10 | 9.23 || 2.03 | 8.21	1337.94 | 26.48 || 923.75 | 31.57	0.00 | 0.35 || 0.0 | 0.27	1.00 | 0.73 || 1.00 | 0.79
	Blue	15.88 | 9.36 || 14.97 | 8.49	1084.76 | 26.48 || 862.05 | 31.57	0.01 | 0.35 || 0.02 | 0.27	0.99 | 0.73 || 0.99 | 0.78
	Orange	0.95 | 9.31 || 1.87 | 8.63	2343.59 | 26.48 || 1524.24 | 31.58	0.00 | 0.35 || 0.00 | 0.27	1.00 | 0.72 || 1.00 | 0.79
	Purple	1.08 | .931 || 1.82 | 8.54	1523.21 | 26.48 || 1027.68 | 31.57	0.00 | 0.35 || 0.00 | 0.27	1.00 | 0.73 || 1.00 | 0.79
	Black	15.53 | 9.35 || 14.47 | 8.60	1084.76 | 26.48 || 862.05 | 31.57	0.01 | 0.35 || 0.02 | 0.27	0.99 | 0.72 || 0.99 | 0.79
	Pink	1.10 | 9.28 || 1.84 | 8.40	1951.16 | 26.48 || 1236.56 | 31.58	0.00 | 0.35 || 0.00 | 0.27	1.00 | 0.73 || 1.00 | 0.79
	Barred	1.17 | 9.26 || 2.12 | 8.58	1555.78 | 26.48 || 998.79 | 31.58	0.00 | 0.35 || 0.00 | 0.27	1.00 | 0.73 || 1.00 | 0.79
	Bald	17.91 | 10.65 || 16.80 | 10.06	1084.76 | 26.48 || 862.05 | 31.57	0.02 | 0.40 || 0.02 | 0.32	0.99 | 0.69 || 0.99 | 0.78
	UV radiance	2.82 | 3.78 || 8.80 | 12.21	1.83 | 1.51 || 3.19 | 2.76	1.54 | 2.51 || 2.76 | 4.42	0.12 | 0.01 || 0.01 | 0.00
	Covered habitat	-0.24 | 0.01 || -0.25 | -0.23	0.14 | 0.05 || 0.15 | 0.11	-1.69 | 0.27 || -1.68 | -2.06	0.09 | 0.79 || 0.09 | 0.04
**feather + colonial + max temperature + total precipitation (Bacterial protection) male | female, complete tree || molecular tree only**					
	Intercept	-14.51 | -9.28 || -15.56 | -17.69	312.39 | 26.33 || 599.93 | 1838.59	-0.05 | -0.35 || -0.03 | 0.00	0.96 | 0.72 || 0.98 | 0.99
	White	14.76 | 9.97 || 15.65 | 17.63	312.39 | 26.32 || 599.93 | 1838.58	0.05 | 0.38 || 0.03 | 0.00	0.96 | 0.70 || 0.98 | 0.99
	Grey	13.64 | 9.31 || 14.35 | 16.53	312.39 | 26.33 || 599.93 | 1838.58	0.04 | 0.35 || 0.02 | 0.00	0.97 | 0.72 || 0.98 | 0.99
	Brown	13.59 | 9.24 || 14.37 | 16.43	312.39 | 26.33 || 599.93 | 1838.58	0.04 | 0.35 || 0.02 | 0.00	0.97 | 0.73 || 0.98 | 0.99

	Green	3.20 | 8.81 || 2.46 | 0.44	313.30 | 26.32 || 603.47 | 1910.21	0.01 | 0.33 || 0.00 | 0.00	0.99 | 0.74 || 1.00 | 0.99
	Yellow	2.75 | 8.87 || 1.94 | 0.03	326.42 | 26.33 || 643.68 | 2921.19	0.01 | 0.34 || 0.00 | 0.00	0.99 | 0.74 || 1.00 | 0.99
	Blue	13.91 | 9.06 || 14.52 | 16.60	312.39 | 26.33 || 599.93 | 1838.58	0.04 | 0.34 || 0.02 | 0.00	0.96 | 0.73 || 0.98 | 0.99
	Orange	3.62 | 9.53 || 3.01 | 0.89	354.10 | 26.33 || 785.70 | 4985.36	0.01 | 0.6 || 0.00 | 0.00	0.99 | 0.72 || 1.00 | 1.00
	Purple	3.28 | 9.09 || 2.48 | 0.23	329.07 | 26.33 || 666.36 | 4617.50	0.01 | 0.35 || 0.00 | 0.00	0.99 | 0.73 || 1.00 | 1.00
	Black	13.46 9.24| || 13.92 | 16.46	312.39 | 26.33 || 599.93 | 1838.58	0.04 | 0.35 || 0.02 | 0.00	0.97 | 0.73 || 0.98 | 0.99
	Pink	2.26 | 8.39 || 1.22 | -0.67	393.47 | 26.34 || 991.19 | 6152.90	0.01 | 0.32 || 0.00 | 0.00	1.00 | 0.75 || 1.00 | 1.00
	Barred	3.30 | 9.35 || 2.58 | 0.50	331.22 | 26.33 || 666.49 | 2993.86	0.01 | 0.36 || 0.00 | 0.00	0.99 | 0.72 || 1.00 | 1.00
	Bald	15.33 | 10.92 || 16.07 | 18.21	312.39 | 26.33 || 599.93 | 1838.58	0.05 | 0.41 || 0.03 | 0.00	0.96 | 0.68 || 0.98 | 0.99
	Colonial	0.10 | 0.04 || 0.33 | 0.17	0.12 | 0.07 || 0.14 | 0.18	0.86 | 0.53 || 2.37 | 0.97	0.39 | 0.60 || 0.02 | 0.33
	Max temperature	0.23 | 0.11 || -0.10 | 0.27	0.22 | 0.11 || 0.14 | 0.26	1.03 | 0.96 || -0.69 | 1.07	0.30 | 0.34 || 0.49 | 0.28
	Total precipitation	-0.41 | -0.48 || 0.19 | -0.35	0.37 |0.19 || 0.17 | 0.30	-1.12 | -2.57 || 1.12 | -1.16	0.26 | 0.01 || 0.26 | 0.25
**max temperature + total precipitation (Thermoregulation) male | female, complete tree || molecular tree only**					
	Intercept	-1.53 | -1.67 || -1.52 | -1.53	0.37 | 0.37 || 0.37 | 0.39	-4.11 | -4.51 || -4.11 | -3.95	0.00 | 0.00 || 0.00 | 0.00
	Max temperature	-0.27 | -0.18 || -0.34 | -0.26	0.41 | 0.43 || 0.43 | 0.42	-0.65 | -0.41 || -0.78 | -0.62	0.52 | 0.68 || 0.43 | 0.54
	Total precipitation	-1.84 | -1.77 || -1.40 | -1.33	0.70 | 0.72 || 0.56 | 0.54	-2.61 | -2.46 || -2.51 | -2.47	0.01 | 0.01 || 0.01 | 0.01

Supplementary Table 4. The results of phylogenetically controlled logistic regressions used to explore five working hypotheses for the function of black skin. These hypotheses are: 1) Gloger's rule (which predicts more black skin at low latitudes; 2) UV protection 1(which predicts black skin in birds that are more exposed to UV radiance and have less protection because of the absence or only lightly coloured feathers); 3) UV protection 2 (which predicts, in addition to the factors of UV protection 1, that open habitats are expected to be more associated with black skin compared to closed habitats as a result of exposure to UV radiance); 4) Bacterial protection hypothesis (which predicts that lightly coloured feathers, high temperatures, high moisture levels and a colonial lifestyle are more prone to have black skin as a form of bacterial protection) and 5) Thermoregulation (which predicts more black skin in birds living in cold and dry areas). Statistics (variable estimates and standard errors, as well as *Z*-value and corresponding p-value for two-sided tests without multiple comparison adjustments) for male and female datasets of all passerines for both the complete and the molecular only phylogenetic tree.

**Model**	**Factor**	**Estimate**	**SE**	***Z*-value**	***p*-value**
**latitude (Gloger's Rule) male | female, complete tree || molecular tree only**					
	Intercept	-2.60 | -2.66 || -2.64 | -2.70	0.26 | 0.28 || 0.29 | 0.29	-9.86 | -9.61 || -9.18 | -9.23	0.00 | 0.00 || 0.00 | 0.00
	Latitude	-1.83 | -1.71 || -1.66 | -1.40	0.80 | 0.80 || 0.84 | 0.82	-2.30 | -2.13 || -1.98 | -1.70	0.02 | 0.03 || 0.05 | 0.09
**feather + UV radiance (UV protection 1) male | female, complete tree || molecular tree only**					
	Intercept	1.42 | -17.48 || -19.59 | -13.91	1.13 | 7.89 || 10.25 | 8.51	1.26 | -2.22 || -1.91 | -1.63	0.21 | 0.03 || 0.06 | 0.10
	White	0.71 | 0.83 || 1.19 | 0.81	0.67 | 0.88 || 0.85 | 1.07	1.05 | 0.94 || 1.39 | 1.07	0.29 | 0.35 || 0.16 | 0.45
	Grey	-1.56 | -0.77 || -0.96 | -0.27	0.63 | 0.75 || 0.78 | 0.89	-2.46 | -1.02 || -1.24 | 0.89	0.01 | 0.31 || 0.21 | 0.76
	Brown	-2.02 | -1.63 || -1.44 | -1.26	0.61 | 0.76 || 0.73 | 0.88	-3.33 | -2.16 || -1.97 | 0.88	0.00 | 0.03 || 0.05 | 0.15
	Green	-2.37 | -2.77 || -14.43 | -1.71	1.04 | 1.39 || 1.13 | 1.30	-2.29 | -2.00 || -1.27 | 1.30	0.02 | 0.05 || 0.21 | 0.19
	Yellow	-1.49 | -1.21 || -0.96 | -1.03	0.66 | 0.81 || 0.80 | 0.96	-2.27 | -1.50 || -1.20 | 0.96	0.02 | 0.13 || 0.23 | 0.28
	Blue	-2.51 | -2.14 || -1.72 | -0.68	0.99 | 1.20 || 1.13 | 1.11	-2.54 | -1.79 || -1.52 | 1.11	0.01 | 0.07 || 0.13 | 0.54
	Orange	-1.43 | -0.80 || -0.58 | 0.05	1.33 | 1.68 || 1.55 | 1.84	-1.07 | -0.47 || -0.38 | 1.84	0.28 | 0.64 || 0.71 | 0.98
	Purple	-1.41 | -0.65 || -0.97 | 0.12	1.58 | 2.09 || 1.81 | 2.00	-0.89 | -0.31 || -0.53 | 2.00	0.37 | 0.76 || 0.59 | 0.95
	Black	-2.50 | -1.71 || -1.78 | -1.59	0.67 | 0.81 || 0.79 | 0.95	-3.71 | -2.13 || -2.25 | 0.95	0.00 | 0.06 || 0.02 | 0.10
	Pink	-0.54 | / || -0.40 | /	2.41 | / || 2.93 | /	-0.22 | / || -0.14 | /	0.82 | / || 0.89 | /
	Barred	-1.56 | -1.19 || -0.78 | -0.12	1.57 | 1.71 || 2.10 | 1.82	-0.99 | -0.70 || -0.34 | 1.82	0.32 | 0.49 || 0.71 | 0.95
	Bald	1.14 | 1.37 || 2.23 | 2.18	0.81 | 0.97 || 0.97 | 1.11	1.41 | 1.42 || 2.32 | 1.11	0.16 | 0.15 || 0.02 | 0.05
	UV radiance	-3.34 | 16.96 || 18.73 | 12.73	1.39 | 8.29 || 10.73 | 8.92	-2.40 | 2.05 || 1.75 | 8.92	0.02 | 0.04 || 0.08 | 0.15
**feather + UV radiance+closed habitat (UV protection 2) male | female, complete tree || molecular tree only**					
	Intercept	1.74 | -18.60 || -20.92 | -14.34	1.11 | 7.64 || 10.68 | 8.29	1.58 | -2.44 || -1.96 | -1.73	0.12 | 0.01 || 0.05 | 0.08
	White	0.71 | 0.87 || 0.97 | 0.86	0.68 | 0.89 || 0.85 | 1.04	1.04 | 0.98 || 1.15 | 0.83	0.30 | 0.33 || 0.25 | 0.41

	Grey	-1.37 | -0.77 || -1.17 | -0.36	0.62 | 0.76 || 0.77 | 0.86	-2.21 | -1.02 || -1.51 | -0.42	0.03 | 0.31 || 0.13 | 0.68
	Brown	-1.99 | -1.57 || -1.69 | -1.39	0.61 | 0.77 || 0.72 | 0.86	-3.26 | -2.05 || -2.34 | -1.61	0.00 | 0.04 || 0.02 | 0.11
	Green	-2.34 | -2.70 || -1.51 | -1.86	1.05 | 1.36 || 1.12 | 1.29	-2.23 | -1.99 || -1.35 | -1.43	0.03 | 0.05 || 0.18 | 0.15
	Yellow	-1.39 | -1.29 || -1.18 | -1.24	0.65 | 0.83 || 0.81 | 0.95	-2.13 | -1.56 || -1.46 | -1.30	0.03 | 0.12 || 0.14 | 0.19
	Blue	-2.60 | -2.14 || -1.84 | -0.98	1.02 | 1.19 || 1.12 | 1.13	-2.54 | -1.80 || -1.64 | -0.87	0.01 | 0.07 || 0.10 | 0.38
	Orange	-1.29 | -0.90 || -0.54 | -0.39	1.30 | 1.67 || 1.51 | 1.97	-0.99 | -0.54 || -0.36 | -0.20	0.32 | 0.59 || 0.72 | 0.84
	Purple	-1.17 | -0.51 || -0.57 | -0.27	1.49 | 2.04 || 1.53 | 2.16	-0.78 | -0.25 || -0.37 | -0.12	0.43 | 0.80 || 0.71 | 0.90
	Black	-2.44 | -1.55 || -2.01 | -1.68	0.68 | 0.80 || 0.79 | 0.93	-3.62 | -1.93 || -2.54 | -1.80	0.00 | 0.05 || 0.01 | 0.07
	Pink	-0.79 | / || -0.51 | /	2.42 | / || 2.69 | /	-0.33 | / || -0.19 | /	0.74 | / || 0.85 | /
	Barred	-1.29 | -1.21 || -0.18 | -0.48	1.50 | 1.69 || 1.76 | 1.95	-0.86 | -0.72 || -0.10 | -0.24	0.39 | 0.47 || 0.92 | 0.81
	Bald	1.18 | 1.51 || 2.07 | 1.89	0.82 | 0.97 || 0.95 | 1.08	1.44 | 1.55 || 2.18 | 1.74	0.15 | 0.12 || 0.03 | 0.08
	UV radiance	-3.99 | 18.05 || 20.12 | 13.24	1.37 | 7.99 || 11.20 | 8.70	-2.91 | 2.26 || 1.80 | 1.52	0.00 | 0.02 || 0.07 | 0.13
	Covered habitat	0.34 | 0.28 || 0.38 | 0.26	0.25 | 0.23 || 0.32 | 0.28	1.35 | 1.21 || 1.19 | 0.94	0.18 | 0.23 || 0.23 | 0.35
**feather + colonial + max temperature + total precipitation (Bacterial protection) male | female, complete tree || molecular tree only**					
	Intercept	-1.80 | -0.94 || 0.24 | 0.69	0.77 | 0.75 || 1.00 | 1.22	-2.32 | -1.25 || 0.24 | 0.57	0.02 | 0.21 || 0.81 | 0.57
	White	0.98 | 0.74 || 0.35 | -0.06	0.69 | 0.83 || 0.51 | 0.57	1.43 | 0.89 || 0.69 | -0.11	0.15 | 0.38 || 0.49 | 0.91
	Grey	-1.23 | -0.86 || -0.29 | -0.36	0.64 | 0.70 || 0.31 | 0.43	-1.92 | -1.24 || -0.94 | -0.84	0.05 | 0.21 || 0.35 | 0.40
	Brown	-1.57 | -1.62 || -0.33 | -0.47	0.62 | 0.73 || 0.31 | 0.44	-2.52 | -2.22 || -1.05 | -1.06	0.01 | 0.03 || 0.29 | 0.29
	Green	-2.04 | -2.99 || -1.01 | -1.39	1.01 | 1.47 || 0.40 | 0.53	-2.03 | -2.04 || -2.53 | -2.60	0.04 | 0.04 || 0.01 | 0.01
	Yellow	-1.14 | -1.39 || -0.25 | -0.53	0.65 | 0.80 || 0.31 | 0.45	-1.74 | -1.75 || -0.80 | -1.17	0.08 | 0.08 || 0.42 | 0.24
	Blue	-2.17 | -1.78 || -0.32 | -0.48	0.98 | 1.06 || 0.32 | 0.46	-2.22 | -1.68 || -0.99 | -1.06	0.03 | 0.09 || 0.32 | 0.29
	Orange	-1.34 | -0.82 || -14.51 | -12.08	1.34 | 1.58 || 466.82 | 161.01	-1.00 | -0.52 || -0.03 | -	0.32 | 0.60 || 0.98 | 0.94
	Purple	-1.18 | -0.43 || -14.35 | -11.88	1.47 | 1.91 || 426.50 | 178.49	-0.80 | -0.23 || -0.03 | -0.07	0.42 | 0.82 || 0.98 | 0.95
	Black	-2.12 | -1.53 || -0.38 | -0.47	0.68 | 0.76 || 0.32 | 0.45	-3.13 | -2.00 || -1.19 | -1.06	0.00 | 0.05 || 0.23 | 0.29
	Pink	-0.30 | / || -14.68 | /	2.47 | / || 1225.55 | /	-0.12 | / || -0.01 | /	0.90 | / || 0.99 | /
	Barred	-1.08 | -1.03 || -14.51 | -12.06	1.42 | 1.58 || 658.41 | 158.51	-0.76 | -0.65 || -0.02 |	0.45 | 0.51 || 0.98 | 0.94
	Bald	1.52 | 1.55 || 0.84 | 0.48	0.83 | 0.93 || 0.76 | 0.81	1.84 | 1.67 || 1.10 | -0.08	0.07 | 0.10 || 0.27 | 0.55
	Colonial	0.11 |0.20 || -0.01 | -0.02	0.36 | 0.33 || 0.07 | 0.06	0.31 | 0.62 || -0.1 | 0.59	0.76 | 0.54 || 0.83 | 0.78
	Max temperature	1.17 | -0.04 || 0.27 | 0.16	0.74 | 0.60 || 0.13 | 0.11	1.57 | -0.07 || 2.10 |	0.12 | 0.94 || 0.03 | 0.16
	Total precipitation	-0.76 | -0.82 || -0.07 | 0.02	0.68 | 0.66 || 0.12 | 0.11	-1.13 | -1.24 || -0.61 | 0.20	0.26 | 0.21 || 0.54 | 0.84
**max temperature + total precipitation (Thermoregulation) male | female, complete tree || molecular tree only**					
	Intercept	-3.97 | -3.66 || -3.90 | -3.51	0.73 | 0.69 || 0.77 | 0.71	-5.45 | -5.27 || -5.08 | -4.67	0.00 | 0.00 || 0.00 | 0.00
	Max temperature	1.32 | 1.37 || 1.43 | 1.03	0.92 | 0.87 || 0.98 | 0.91	1.44 | 1.57 || 1.46 | 1.13	0.15 | 0.12 || 0.14 | 0.26
	Total precipitation	0.23 | -0.90 || -0.32 | -0.65	0.73 | 0.77 || 0.83 | 0.85	0.32 | -1.17 || -0.39 | -0.76	0.75 | 0.24 || 0.70 | 0.44

Supplementary Table 5. The results of phylogenetically controlled logistic regression between black skin and the individual variables used in the more complex models above. Statistics (number of samples used, AIC- and logLik-values, variable estimates and standard errors, as well as *Z*-value and corresponding p-value for two-sided tests without multiple comparison adjustments) are shown for males and females of all birds for both the complete and the molecular only phylogenetic tree.

**Model**	**Factor**	**Estimate**	**SE**	***Z*-value**	***p*-value**
**Feather colour male | female, complete tree || molecular tree only**<br>***n*=2241, AIC=758.5, logLik=-365.2, R^2=0.32 | *n*=2240, AIC=785.72, logLik=-378.9, R^2=0.26 || *n*=1819, AIC=616.5, -logLik=-294.2, R^2=0.30 | , *n*=1819, AIC=658.4, -logLik=-315.2, R^2=0.23**					
	Intercept	-1.81 | -2.23 || -1.69 | -1.78	0.43 | 0.60 || 0.48 | 0.57	-4.15 | -3.73 || -3.54 | -3.14	0.00 | 0.00 || 0.00 | 0.00
	White	1.53 | 1.79 || 1.37 | 1.14	0.48 | 0.64 || 0.51 | 0.59	3.17 | 2.82 || 2.66 | 2.32	0.00 | 0.01 || 0.00 | 0.03
	Grey	-0.81 | -0.11 || -0.72 | -0.19	0.48 | 0.59 || 0.51 | 0.53	-1.68 | -0.18 || -1.42 | -0.36	0.09 | 0.86 || 0.16 | 0.89
	Brown	-1.33 | -0.77 || -1.13 | -0.87	0.46 | 0.58 || 0.50 | 0.54	-2.86 | -1.33 || -2.27 | -1.62	0.00 | 0.18 || 0.02 | 0.57
	Green	-1.88 | -2.31 || -1.78 | -1.93	0.67 | 0.92 || 0.69 | 0.75	-2.79 | -2.52 || -2.60 | -2.59	0.01 | 0.01 || 0.01 | 0.00
	Yellow	-0.89 | -0.36 || -0.84 | -0.72	0.56 | 0.64 || 0.57 | 0.61	-1.59 | -0.55 || -1.47 | -1.17	0.11 |0.58 || 0.14 | 0.02
	Blue	-1.25 | -0.67 || -1.25 | -0.49	0.67 | 0.77 || 0.71 | 0.67	-1.86 | -0.87 || -1.77 | -0.73	0.06 | 0.38 || 0.10 | 0.72
	Orange	-15.25 | -0.32 || -0.80 | -0.51	1596.65 | 1.47 || 1.26 | 1.46	-0.01 | -0.22 || -0.63 | -0.35	0.99 | 0.83 || 0.52 | 0.11
	Purple	-15.25 | -0.45 || 0.91 | -0.52	1400.36 | 1.83 || 1.13 | 1.44	-0.01 | -0.25 || -0.81 | -0.36	0.99 | 0.81 || 0.42 | 0.01
	Black	-1.99 | -0.86 || -2.13 | -1.11	0.53 | 0.61 || 0.58 | 0.57	-4.15 | -1.41 || -3.71 | -2.12	0.00 | 0.16 || 0.00 | 0.24
	Pink	-15.25 | -0.01 || -0.47 | -0.31	2524.53 | 2.09 || 1.83 | 2.25	-3.74 | -0.004 || -0.26 | -0.14	1.00 | 0.99 || 0.80 | 0.46

<table>
<tr><td></td><td>Barred</td><td>-15.25 | -0.74 || -0.84 | -0.67</td><td>1596.65 | 1.33 || 1.17 | 1.17</td><td>-0.01 | -0.55 || -0.72 | -0.57</td><td>0.99 | 0.58 || 0.47 | 0.73</td></tr>
<tr><td></td><td>Bald</td><td>2.25 | 2.57 || 1.96 | 1.91</td><td>0.53 | 0.67 || 0.57 | 0.64</td><td>-0.01 | 3.82 || 3.47 | 2.97</td><td>0.00 | 0.00 || 0.00 | 0.72</td></tr>
<tr><td colspan="6">Feather colour HBW male|female , complete tree || molecular tree only
n=2145, AIC=730.6, logLik=-351.3, R^2=0.13 n=2145, AIC=762.2, logLik=-367.1, R^2=0.27 || n=1735, AIC=713.2, -logLik=-342.6, R^2=0.11 | n=1818, AIC=639.4, logLik=-305.7, R^2=0.24</td></tr>
<tr><td></td><td>Intercept</td><td>-1.83 | -2.03 || -2.58 | -2.08</td><td>0.44 | 0.57 || 0.51 | 0.63</td><td>-4.15 | -3.54 || -5.09 | -3.31</td><td>0.00 | 0.00 || 0.00 | 0.00</td></tr>
<tr><td></td><td>White</td><td>1.53 | 1.76 || 1.21 | 1.81</td><td>0.48 | 0.62 || 0.35 | 0.67</td><td>3.17 | 2.84 || 3.48 | 2.70</td><td>0.00 | 0.00 || 0.00 | 0.01</td></tr>
<tr><td></td><td>Grey</td><td>-0.58 | -0.23 || 0.59 | -0.15</td><td>0.45 | 0.57 || 0.27 | 0.62</td><td>-1.28 | -0.40 || 2.23 | -0.24</td><td>0.20 | 0.69 || 0.03 | 0.81</td></tr>
<tr><td></td><td>Brown</td><td>-1.13 | -0.97 || 0.01 | -0.78</td><td>0.45 | 0.57 || 0.26 | 0.62</td><td>-2.50 | -1.70 || 0.05 | -1.26</td><td>0.01 | 0.09 || 0.96 | 0.21</td></tr>
<tr><td></td><td>Green</td><td>-1.16 | -1.46 || 0.42 | -1.06</td><td>0.54 | 0.69 || 0.34 | 0.70</td><td>-2.16 | -2.12 || 1.23 | -1.51</td><td>0.03 | 0.03 || 0.22 | 0.13</td></tr>
<tr><td></td><td>Yellow</td><td>-0.47 | -1.23 || 0.59 | -1.13</td><td>0.51 | 0.79 || 0.30 | 0.84</td><td>-0.92 | -1.55 || 1.95 | -1.34</td><td>0.36 | 0.12 || 0.05 | 0.18</td></tr>
<tr><td></td><td>Blue</td><td>-1.54 | -1.26 || 0.19 | -0.69</td><td>0.69 | 0.82 || 0.46 | 0.79</td><td>-2.24 | -1.54 || 0.42 | -0.88</td><td>0.02 | 0.12 || 0.68 | 0.38</td></tr>
<tr><td></td><td>Orange</td><td>-0.62 | 0.27 || -13.16 | -0.42</td><td>1.33 | 1.41 || 1302.88 | 1.68</td><td>-0.47 | 0.19 || -0.01 | -0.25</td><td>0.64 | 0.85 || 0.99 | 0.80</td></tr>
<tr><td></td><td>Purple</td><td>-0.71 | 0.75 || -13.16 | 1.20</td><td>1.03 |1.11 || 868.59 | 1.06</td><td>-0.69 |0.68 || -0.02 | 1.13</td><td>0.49 | 0.50 || 0.99 | 0.26</td></tr>
<tr><td></td><td>Black</td><td>-2.12 | -1.55 || 0.96 | -1.47</td><td>0.54 | 0.63 || 0.52 | 0.68</td><td>-3.95 | -2.46 || 1.84 | -2.16</td><td>0.00 | 0.01 || 0.07 | 0.03</td></tr>
<tr><td></td><td>Pink</td><td>-0.50 | 0.24 || -13.16 | -0.09</td><td>1.72 | 1.78 || 1504.44 | 2.38</td><td>-0.29 | 0.13 || -0.01 | -0.04</td><td>0.77 | 0.89 || 0.99 | 0.97</td></tr>
<tr><td></td><td>Barred</td><td>-0.64 | -0.49 || 0.80 | -0.30</td><td>1.13 | 1.18 || 0.49 | 1.37</td><td>-0.57 | -0.41 || 1.64 | -0.22</td><td>0.57 | 0.68 || 0.10 | 0.83</td></tr>
<tr><td></td><td>Bald</td><td>2.35 | 2.26 || 3.53 | 2.10</td><td>0.53 | 0.65 || 1.09 | 0.71</td><td>4.39 | 3.46 || 3.25 | 2.95</td><td>0.00 | 0.00 || 0.00 | 0.00</td></tr>
<tr><td colspan="6">UV radiance male | female , complete tree || molecular tree only
n=2228, AIC=969.3, -logLik=-481.6, R^2=0.07 | n=2228, AIC=914.1, logLik=-453.7, R^2=0.08 || n= 1809, AIC=755.6, -logLik= -374.8, R^2=0.06 | n=1809, AIC=749.2, -logLik=-371.6, R^2=0.06</td></tr>
<tr><td></td><td>Intercept</td><td>-9.72 | -8.66 || -4.87 | -4.68</td><td>2.14 | 4.34 || 0.73 | 0.72</td><td>-4.55 | -1.95 || -6.63 | 0.72</td><td>0 | 0.05 || 0.00 | 0.00</td></tr>
<tr><td></td><td>UV radiance</td><td>0.99 | 6.26 || 3.43 | 3.09</td><td>0.22 | 4.67 || 1.07 | 1.07</td><td>4.56 | 1.34 || 3.20 | 1.07</td><td>0.00 | 0.18 || 0.00 | 0.00</td></tr>
<tr><td colspan="6">Dichromatism male | female , complete tree || molecular tree only
n=2242, AIC=944.6, logLIk=-469.3, R^2=0.07 | n=2242, AIC=917.3, logLik=-455.7, R^2= 0.08 || n=1819, AIC=765.4, -logLik=-379.7, R^2=0.05 | n=1818, AIC=751.7, -logLik=-372.9, R^2=0.06</td></tr>
<tr><td></td><td>Intercept</td><td>-2.58 | -2.62 || -2.38 | -2.33</td><td>0.15 | 0.16 || 0.18 | 0.19</td><td>-16.81 | -16.44 || -13.05 | -12.46</td><td>0.00 | 0.00 || 0.00 | 0.00</td></tr>
<tr><td></td><td>Present</td><td>-0.31 | -0.42 || -0.47 | -0.66</td><td>0.20 | 0.21 || 0.21 | 0.22</td><td>-1.57 | -2.02 || -2.28 | -3.02</td><td>0.12 | 0.04 || 0.02 | 0.00</td></tr>
<tr><td colspan="6">Colonial male | female , complete tree || molecular tree only
n=2242, AIC=931.7, logLik=-462.8, R^2=0.08 | n=2242, AIC=906.8, logLik=-450.4, R^2=0.09 || n=1819, AIC=758.6, -logLik=-376.3, R^2=0.06 | n=1818, AIC=748.1, -logLik=-371.1, R^2=0.07</td></tr>
<tr><td></td><td>Intercept</td><td>-2.94 | -2.96 || -2.87 | -2.89</td><td>0.17 | 0.18 || 0.19 | 0.19</td><td>-17.02 | -16.60 || -15.32 | -14.90</td><td>0.00 | 0.00 || 0.00 | 0.00</td></tr>
<tr><td></td><td>Yes</td><td>0.93 | 0.93 ||0.82 | 0.84</td><td>0.22 | 0.23 || 0.24 | 0.24</td><td>4.15 | 4.10 || 3.42 | 3.50</td><td>0.00 | 0.00 || 0.00 | 0.00</td></tr>
<tr><td colspan="6">Closed habitat male | female , complete tree || molecular tree only
n=2242, AIC=945.4, logLik=-469.7, R^2=0.06 | n=2242, AIC=922.1, logLik=-458.0, R^2=0.08 || n=1819, AIC=767.3, logLik=-380.6, R^2=0.05 | n=1818, AIC=761.1, -logLik=-377.5, R^2=0.05</td></tr>
<tr><td></td><td>Intercept</td><td>-2.63 | -2.67 || -2.57 | -2.69</td><td>0.15 | 0.15 || 0.17 | 0.16</td><td>-17.51 | -17.36 || -15.19 | -16.71</td><td>0.00 | 0.00 || 0.00 | 0.00</td></tr>
<tr><td></td><td>Yes</td><td>-0.26 | -0.19 || -0.34 | -0.16</td><td>0.19 | 0.19 || 0.21 | 0.26</td><td>-1.38 | -1.01 || -1.62 | -0.61</td><td>0.17 | 0.31 || 0.10 | 0.54</td></tr>
<tr><td colspan="6">Age male | female, complete tree || molecular tree only
n=2110, AIC=864, logLik=-429.0, R^2=0.08 | n=2111, AIC=854.8, logLik=-424.4, R^2=0.08 || n=1720, AIC=697.9, -logLik=-346.0, R^2=0.05 | n=1731, AIC=692.9, -logLik=-343.4, R^2=0.05</td></tr>
<tr><td></td><td>Intercept</td><td>-2.64 | -2.72 || -2.72 | -2.79</td><td>0.56 | 0.44 || 0.54 | 0.48</td><td>-4.74 | -6.24 || -5.08 | -5.83</td><td>0.00 | 0.00 || 0.00 | 0.00</td></tr>
<tr><td></td><td>Age</td><td>-1.16 | -0.52 || -0.23 | 0.09</td><td>5.23 | 3.97 || 4.91 | 4.33</td><td>-0.22 | -0.13 || -0.05 | 0.02</td><td>0.83 | 0.90 || 0.96 | 0.98</td></tr>
<tr><td colspan="6">Mass male | female, complete tree || molecular tree only
n=2124, AIC=876.3, logLik=-435.1, R^2=0.10 | n=2124, AIC=866.6, loglik=-430.3, R^2=0.11 || n=1758, AIC=719.2, -logLik=-356.6, R^2=0.09 | n=1757, AIC=721.2, -logLik=-357.6, R^2=0.08</td></tr>
<tr><td></td><td>Intercept</td><td>-3.80| -3.95 || -0.19 | -0.22</td><td>0.42 | 0.46 || 0.43 | 0.43</td><td>-8.95 | -8.59 || -0.45 | -0.52</td><td>0.00 | 0.00 || 0.66 | 0.60</td></tr>
<tr><td></td><td>Mass</td><td>2.76 | 3.20 || 0.24 | 0.24</td><td>0.83 | 0.85 || 0.09 | 0.09</td><td>3.34 | 3.76 || 2.82 | 2.81</td><td>0.00 | 0.00 ||0.00 | 0.01</td></tr>
<tr><td colspan="6">Total precipitation male | female, complete tree || molecular tree only
n=2232, AIC=939.6, logLik=-466.8, R^2=0.07 | n=2233, AIC=915.9, logLik= -454.9, R^2=0.08 || n=1808, AIC=765.0, -logLik=-379.5, R^2=0.05 | n=1808n AIC=753.8, -logLik=-373.9, R^2=0.05</td></tr>
<tr><td></td><td>Intercept</td><td>-2.32 | -2.38 || -2.27 |</td><td>0.19 | 0.20 || 0.21 | -2.61</td><td>-12.03 | -11.92 || -10.66 | -12.13</td><td>0.00 | 0.00 || 0.00 | 0.00</td></tr>
<tr><td></td><td>Total precipitation</td><td>-1.37 | -1.34 || -1.24 |</td><td>0.55 | 0.56 || 0.57 | -0.04</td><td>-2.50 | -2.42 || -2.18 | -1.06</td><td>0.01 | 0.02 || 0.03 | 0.29</td></tr>
<tr><td colspan="6">Maximum temperature male| female, complete tree || molecular tree only
n=2233, AIC=930.4, logLik=-462.2, R^2=0.07 | n=2231, AIC=913.5, logLik=-453.8, R^2=0.08 || n=1808, AIC=798.3, -logLik=-396.2, R^2=0.04 | R^2=0.00 | n=1808, AIC=751.2, -logLik= -372.6, R^2= 0.05</td></tr>
<tr><td></td><td>Intercept</td><td>-3.38 | -3.39 || -2.96 | -2.09</td><td>0.39 | 0.40 || 0.71 | 0.26</td><td>-8.64 | -8.58 || -4.16 | -7.93</td><td>0.00 | 0.00 || 0.00 | 0.00</td></tr>
<tr><td></td><td>Max. Temp.</td><td>1.00 | 0.96 || 0.05 | -0.96</td><td>0.50 | 0.50 || 0.01 | 0.37</td><td>2.02 | 1.92 || 5.01 | -2.58</td><td>0.05 | 0.06 || 0.00 | 0.01</td></tr>
</table>

Supplementary Table 6. The results of phylogenetically controlled logistic regression between black skin and the individual variables used in the more complex models above. Statistics (number of samples used, AIC- and logLik-values, variable estimates and standard errors, as well as *Z*-value and corresponding p-value for two-sided tests without multiple comparison adjustments) are shown for males and females of all non-passerines for both the complete and the molecular only phylogenetic tree.

<table>
<tr><th>Model</th><th>Factor</th><th>Estimate</th><th>SE</th><th>Z-value</th><th>p-value</th></tr>
<tr><td colspan="6">Feather colour male| female, complete tree || molecular tree only
n=974, AIC=338.2, logLik=-155.1, R^2=0.42 | n=969, AIC=351.7, logLik=-161.9, R^2=0.33 || n=781, AIC=290.2, -logLik=-131.1, R^2=0.41 | n=780, AIC=337.2, -logLik=-154.6, R^2=0.28</td></tr>
<tr><td></td><td>Intercept</td><td>-0.49| -16.49 || -0.53 | 0.12</td><td>1.33| 921.77 || 1.65 | 1.73</td><td>-0.37| -0.02 || -0.32 | 0.07</td><td>0.71| 0.99 || 0.75 | 0.94</td></tr>
</table>

	White	0.53| 16.80 || 0.64 | 0.64	0.52| 921.76 || 0.62 | 0.44	1.02| 0.02 || 1.03 | 1.44	0.31| 0.99 || 0.30 | 0.15
	Grey	-0.48| 15.61 || -0.27 | -0.02	0.61| 921.76 || 0.46 | 0.11	-0.80| 0.02 || -0.58 | -0.20	0.42| 0.99 || 0.56 | 0.84
	Brown	-0.59| 15.55 || -0.38 | -0.09	0.72| 921.76 || 0.58 | 0.11	-0.81| 0.02 || -0.66 | -0.79	0.41|0 .99 || 0.51 | 0.43
	Green	-14.71| 1.70 || -13.88 | -0.96	167.65| 931.87 || 125.48 | 0.45	-0.09| 0.00 || -0.11 | -2.17	0.93| 1.00 || 0.91 | 0.03
	Yellow	-14.71| 1.70 || -13.88 | -0.13	533.93| 1070.12 || 359.63 | 0.20	-0.03| 0.00 || -0.04 | -0.68	0.98| 1.00 || 0.97 | 0.50
	Blue	-0.74| 15.66 || -0.04 | -0.01	0.84| 921.76 || 0.42 | 0.21	-0.88| 0.02 || -0.09 | -0.04	0.38| 0.99 || 0.93 | 0.96
	Orange	-14.71| 1.69 || -13.88 | -0.13	0.13| 1476.30 || 1345.61 | 0.048	-0.23| 0.00 || -0.01 | -0.26	0.82| 1.00 || 0.99 | 0.79
	Purple	-14.71| 1.69 || -13.88 | -0.13	1412.63| 1476.27 || 601.77 | 0.33	-0.01| 0.00 || -0.02 | -0.38	0.99| 1.00 || 0.98 | 0.70
	Black	-0.65| 15.57 || -0.42 | -0.13	755.08| 921.76 || 0.62 | 0.18	-0.02| 0.0 || -0.67 | -0.71	0.98| 0.99 || 0.50 | 0.48
	Pink	-14.71| 1.69 || -13.88 | -0.08	1153.41| 1317.64 || 951.49 |0.52	-0.01| 0.00 || -0.01 | -0.15	0.99| 1.00 || 0.99 | 0.88
	Barred	-14.71| 1.69 || -13.88 | -0.13	755.08| 1108.88 || 601.77 | 0.23	-0.02| 0.00 || -0.02 | -0.56	0.99| 1..00 || 0.98 | 0.58
	Bald	0.95| 17.34 || 1.28 | 0.73	0.68 | 921.76 || 0.89 | 1.07	1.39| 0.02 || 1.45 | 0.68	0.16| 0.99 || 0.15 | 0.49
**Feather colour HBW male | female, complete tree || molecular tree only** **_n_=881, AIC=309.4, logLik=-140.7, R^2=0.16 | _n_=881, AIC=339.1, logLik=-155.5, R^2=0.35 || _n_=701, AIC=340.1, -logLik=-156.1, R^2=0.18 | _n_=707, AIC=292.9, -logLik=132.5, R^2=0.36**					
	Intercept	-1.01| -10.24 || 0.18 | -16.17	2.05| 41.92 || 1.65 | 936.83	-0.49| -0.24 || 0.11 | -0.02	0.62| 0.81 || 0.91 | 0.99
	White	1.04| 10.83 || 0.27 | 16.15	1.02| 41.91 || 0.23 | 936.83	1.02| 0.26 || 1.13 | 0.02	0.31| 0.80 || 0.26 | 0.99
	Grey	0.03| 10.05 || -0.03 | 15.19	0.24| 41.91 || 0.12 | 936.83	0.13| 0.24 || -0.26 | 0.02	0.90| 0.81 || 0.80 | 0.99
	Brown	-0.03| 10.02 | |-0.07 | 15.20	0.23 | 41.91 || 0.11 | 936.83	-0.14| 0.24 || -0.66 | 0.02	0.89| 0.81 || 0.51 | 0.99
	Green	-13.32| 9.43 || -0.10 | 2.83	106.41| 41.91 || 0.14 | 936.83	-0.13| 0.22 || -0.69 | 0.00	0.90| 0.82 || 0.49 | 1.00
	Yellow	-13.32| 9.96 || -0.04 | 2.83	373.68| 41.91 || 0.20 | 969.38	-0.04| 0.24 || -0.18 | 0.00	0.97| 0.81 || 0.86 | 1.00
	Blue	-0.08| 9.94 || -14.05 | 15.26	0.28| 41.91 || 248.51 | 936.83	-0.27| 0.24 || -0.06 | 0.02	0.79| 0.81 || 0.95 | 0.99
	Orange	-13.32| 9.99 || -14.05 | 2.83	1294.48|41.92 || 724.53 | 1224.00	-0.01| 0.24 || -0.02 | 0.00	0.99| 0.81 || 0.98 | 1.00
	Purple	-13.32| 10.55 || -14.05 | 16.58	489.27| 41.91 || 418.31 | 936.83	-0.03| 0.25 || -0.03 | 0.02	0.98| 0.80 || 0.97 | 1.00
	Black	-0.14| 10.01 || -0.03 |15.14	0.32| 41.91 || 0.22 | 936.83	-0.45| 0.24 || -0.14 | 0.02	0.65| 0.81 || 0.89 | 0.99
	Pink	-13.32| 9.97 || -14.05 | 2.83	747.37| 41.92 || 724.53 | 1089.91	-0.02| 0.24 || -0.02 | 0.00	0.99| 0.81 || 0.98 | 1.00
	Barred	-13.32| 9.99 || -0.16 | 2.83	915.34| 41.92 || 0.18 | 1089.92	-0.01| 0.24 || -0.92 | 0.00	0.99| 0.81 || 0.36 | 1.00
	Bald	1.58| 11.96 || 0.90 | 16.52	1.25| 41.91 || 1.18 | 936.83	1.26| 0.29 || 0.76 | 0.02	0.21| 0.78 || 0.45 | 0.99
**UV radiance male | female, complete tree || molecular tree only** **_n_=970, AIC=465.2, logLik=-229.6, R^2=0.05 | _n_=969, AIC=460.8, logLik=-227.4, R^2=0.06 || _n_=777, AIC=393.3, -logLik=-193.6, R^2=0.05 | _n_=777, AIC=393, -logLik=-193.5, R^2=0.05**					
	Intercept	-3.20 | -4.26 || -5.26 | -4.88	4.73 | 4.83 || 1.07 | 1.03	-0.68| -0.88 || -4.92 | -4.74	0.50 | 0.38 || 0.00 | 0.00
	UV radiance	0.78 | 1.90 || 4.01 | 3.38	5.01| 5.11 || 1.55 | 1.48	0.16 | 0.37 || 2.58 | 2.29	0.88 | 0.71 || 0.01 | 0.02
**Dichromatism male | female , complete tree || molecular tree only** **_n_=974, AIC=460.3, logLik=-227.1, R^2=0.05 | _n_=974, AIC=455.2, logLik=-224.6, R^2=0.06 | _n_=781, AIC=388.8, -logLik=-191.4, R^2=0.06 | _n_=780, AIC=380.7, -logLik=-187.3, R^2=0.09**					
	Intercept	-2.06 | -2.05 || -1.83 | -1.81	0.26 | 0.27 || -0.38 | 0.39	-7.96 | -7.70 || -4.89 | -4.62	0.00 | 0.00 | 0.00 | 0.00
	Yes	-1.10 | -0.74 || -1.05 | -1.14	0.99 | 0.28 || -0.32 | 0.33	-1.12 | -2.67 || -3.29 | -3.44	0.01 | 0.01 | 0.00 | 0.00
**Colonial male | female, complete tree || molecular tree only** **_n_=969, AIC=443.5, logLik=-218.8, R^2=0.09 | _n_=969, AIC=441.3, logLik=-217.7, R^2=0.09 | _n_=781, AIC=381.2, -logLik=-187.6, R^2=0.09 | _n_=780, AIC=378.2, -logLik=-186.1, R^2=0.10**					
	Intercept	-2.54 | 1.1 || -2.61 | -2.55	0.39 | 0.25 || 0.36 | 0.39	-6.60 | 4.37 || -7.27 | -6.60	0.00 | 0.00 | 0.00 | 0.00
	Yes	1.01 | 0.07 || 1.11 | 1.06	0.31 | 0.03 || 0.32 | 0.32	3.30 | 2.53 || 3.43 | 3.25	0.00 | 0.01 | 0.00 | 0.00
**Closed habitat male | female, complete tree || molecular tree only** **_n_=969, AIC=463.0, logLik=-228.5, R^2=0.04 | _n_=969, AIC=459, logLik=-226.6 | _n_=781, AIC=398.9, -logLik=-196.4, R^2=0.03 | _n_=780, AIC=398.1, -logLik=-196.0, R^2=0.03**					
	Intercept	-2.28 | -2.28 || -2.13 | -2.27	0.21 | 0.22 || 0.24 | 0.22	-10.64 | -10.42 || -9.05 | -10.47	0.00 | 0.00 || 0.00 | 0.00
	Yes	-0.47 | -0.43 || -0.72 | -0.56	0.27 | 0.03 || 0.31 | 0.36	-1.74 | -1.63 || -2.32 | -1.54	0.08 | 0.10 || 0.02 | 0.12
**Age male|female , complete tree || molecular tree only** **_n_=903, AIC=407.8, logLik=-200.9, R^2=0.05 | _n_=900, AIC=403, logLik=-198.5, R^2=0.07 | _n_=729, AIC=349.1, -logLik=-171.5 | _n_=729, AIC=3399, -logLik=-167.0, R^2=0.05**					
	Intercept	-2.55 | -2.64 || -2.59 | 0.00	0.43 | 0.46 || 0.46 | 0.00	-5.87 | -5.78 || -5.58 | 0.00	0.00 | 0.00 || 0.00 | 0.00
	Age	0.13 | 0.07 || 0.07 | 0.00	3.65 | 3.90 || 3.92 | 0.00	0.04 | 0.02 || 0.02 | 0.00	0.97 | 0.99 || 0.99 | 0.99
**Mass male | female, complete tree || molecular tree only** **_n_=941, AIC=415.6, logLik=-204.8, R^2=0.14 | _n_=936, AIC=415.7, logLik=-204.9, R^2=0.14 || _n_=762, AIC=356.5, -logLik=-175.2, R^2=0.14 | _n_=761, AIC=357.6, -logLik=-175.8**					
	Intercept	-4.69 | -4.79 || 0.15 | 0.11	0.79 | 0.70 || 0.48 | 0.49	-5.98 | -6.82 || 0.30 | 0.24	0.00 | 0.00 || 0.76 | 0.81
	Mass	4.71 | 4.69 || 0.30 | 0.29	1.18 | 1.13 || 0.11 | 0.11	1.18 | 4.14 || 2.69 | 2.66	0.00 | 0.00 || 0.01 | 0.01
**Total precipitation male | female, complete tree || molecular tree only** **_n_=970, AIC=452.8, logLik=-223.4, R^2=0.05 | _n_=970, AIC=452.9, logLik=-223.4, R^2=0.07 || _n_=778, AIC=390.9, -logLik=-192.5, R^2=0.06 | _n_=776, AIC=395.2, -logLik=-194.6, R^2=0.03**					
	Intercept	-1.72 | -1.82 || -1.30 | -2.23	0.30 | 0.27 || 0.47 | 0.27	-5.65 | -6.67 || -2.73 | -8.34	0.00 | 0.00 || 0.01 | 0.00
	Total precipitation	-1.87 | -1.87 || -0.15 | -0.89	0.68 | 0.71 || 0.05 | 0.75	-2.75 | -2.64 || -2.99 | -1.20	0.01 | 0.01 || 0.00 | 0.23
**Maximum temperature male|female, complete tree || molecular tree only** **_n_=970, AIC=454.7, logLik=-224.3, R^2=0.05 | _n_=969, AIC=472.7, logLik=-233.4, R^2=0.01 || _n_=778, AIC=395.1, -logLik=-194.5, R^2=0.04 | _n_=776, AIC=394.9, -logLik=-194.4, R^2=0.03**					
	Intercept	-3.25 | -2.68 || -1.74 | -1.93	0.51 | 0.87 || 0.32 | 0.35 |	-6.38 | -3.09 || -5.38 | -5.44	0.00 | 0.00 || 0.00 | 0.00
	Max. Temp.	1.06 | 0.04 || -0.91 | -0.87	0.64 | 0.01 || 0.43 | 0.49 |	-1.67 | -3.76 || -2.14 | -1.77	0.09 | 0.00 || 0.03 | 0.08

Supplementary Table 7. The results of phylogenetically controlled logistic regression between black skin and the individual variables used in the more complex models above. Statistics (number of samples used, AIC- and logLik-values, variable estimates and standard errors, as well as *Z*-value and corresponding p-value for two-sided tests without multiple comparison adjustments) are shown for males and females of all passerines for both the complete and the molecular only phylogenetic tree.

<table>
<tr><th>Model</th><th>Factor</th><th>Estimate</th><th>SE</th><th>Z-value</th><th>p-value</th></tr>
<tr><td colspan="6">Feather colour male | female, complete tree || molecular tree only
n=1267, AIC=438.0, logLik=-205.0, R^2=0.25 | n=1266, AIC=435.5, logLik=-204.9, R^2=0.21 || n=1038, AIC=338.1, -logLik=-155.0, R^2=0.22 | n=1038, AIC=359.8, -logLik=-167.9, R^2=0.13</td></tr>
<tr><td></td><td>Intercept</td><td>-1.50 | -1.59 || -1.81 | -3.27</td><td>0.53 | 0.70 || 0.66 | 0.50</td><td>-2.81 | -2.25 || -2.74 | -6.61</td><td>0.005 | 0.02 || 0.01 | 0.00</td></tr>
<tr><td></td><td>White</td><td>1.35 | 1.37 || 1.45 | 2.76</td><td>0.67 | 0.87 || 0.82 | 0.76</td><td>2.00 | 1.58 || 1.77 | 3.66</td><td>0.05 | 0.11 || 0.08 | 0.00</td></tr>
<tr><td></td><td>Grey</td><td>-1.26 | -0.70 || -1.09 | 0.86</td><td>0.65 | 0.75 || 0.78 | 0.52</td><td>-1.95 |-0.94 || -1.41 | 1.65</td><td>0.05 | 0.35 || 0.16 | 0.10</td></tr>
<tr><td></td><td>Brown</td><td>-1.65 | -1.42 || -1.56 | 0.04</td><td>0.60 | 0.75 || 0.73 | 0.46</td><td>-2.74 | -1.90 || -2.14 | 0.08</td><td>0.006 | 0.06 || 0.03 | 0.93</td></tr>
<tr><td></td><td>Green</td><td>-1.80 | -2.27 || -1.59 | -0.53</td><td>0.98 | 1.23 || 1.18 | 1.15</td><td>-1.85 | -1.84 || -1.35 | -0.46</td><td>0.06 | 0.07 || 0.18 | 0.64</td></tr>
<tr><td></td><td>Yellow</td><td>-1.08 | -1.13 || -1.16 | 0.05</td><td>0.66 | 0.82 || 0.83 | 0.70</td><td>-1.63 | -1.39 || -1.39 | 0.08</td><td>0.10 | 0.17 || 0.17 | 0.94</td></tr>
<tr><td></td><td>Blue</td><td>-2.12 | -1.81 || -1.88 | 0.28</td><td>1.01 | 1.15 || 1.22 | 0.95</td><td>-2.10 | -1.57 || -1.54 | 0.29</td><td>0.04 | 0.12 || 0.12 | 0.77</td></tr>
<tr><td></td><td>Orange</td><td>-1.15 |-14.65 || -0.53 | 1.47</td><td>1.44 | 1675.59 || 1.55 | 1.69</td><td>-0.80 | -0.01 || -0.34 | 0.87</td><td>0.43 | 0.99 || 0.73 | 0.34</td></tr>
<tr><td></td><td>Purple</td><td>-0.83 | -14.65 || -0.45 | 1.68</td><td>1.57 | 2369.65 || 1.58 | 1.91</td><td>-0.53 | -0.01 || -0.29 | 0.88</td><td>0.60 | 1.00 || 0.77 | 0.38</td></tr>
<tr><td></td><td>Black</td><td>-1.90 | -1.54 || -1.95 | 1.39</td><td>0.65 | 0.80 || 0.80 | 0.92</td><td>-2.91 | -1.93 || -2.39 | 1.51</td><td>0.004 | 0.05 || 0.17 | 0.13</td></tr>
<tr><td></td><td>Pink</td><td>0.26 | / || 0.29 | /</td><td>2.43 | / || 2.66 | /</td><td>0.11 | / || 0.11 | /</td><td>0.91 | / || 0.91 | /</td></tr>
<tr><td></td><td>Barred</td><td>-0.39 | -14.65 || -0.07 | 1.42</td><td>1.68 |1675.59 || 1.77 | 1.70</td><td>-0.23 | -0.01 ||-0.04 | 0.83</td><td>0.81 | 0.99 || 0.97 | 0.41</td></tr>
<tr><td></td><td>Bald</td><td>1.98 | 1.88 || 2.33 | /</td><td>0.81 |0.97 || 0.93 | /</td><td>2.43 | 1.95 || 2.52 | /</td><td>0.02 | 0.05 || 0.01 | /</td></tr>
<tr><td colspan="6">Feather colour HBW male | female, complete tree || molecular tree only
n=1264, AIC=426, logLik=- 199.0, R^2=0.15 | n=1264, AIC=436.5, logLik=-205.2, R^2=0.35 || n=1034, AIC=375.0, -logLik=-173.5, R^2=0.11 | n=1035, AIC=348.0, -logLik=-161.0, R^2=0.17</td></tr>
<tr><td></td><td>Intercept</td><td>-1.72 | 1.57 || -3.81 | -1.74</td><td>0.53 | 0.70 || 0.62 | 0.78</td><td>-3.22 | -2.24 || -6.17 | -2.25</td><td>0.00 | 0.03 || 0.00 | 0.02</td></tr>
<tr><td></td><td>White</td><td>1.49 | 1.38 || 1.28 | 1.33</td><td>0.68 | 0.87 || 0.68 | 0.97</td><td>2.20 | 1.58 || 1.88 | 1.37</td><td>0.03 | 0.11 || 0.06 | 0.17</td></tr>
<tr><td></td><td>Grey</td><td>-1.18 | -0.91 || 0.97 | -0.74</td><td>0.64 | 0.75 || 0.64 | 0.82</td><td>-1.86 | -1.22 || 1.52 | -0.89</td><td>0.06 | 0.2 2 || 0.13 | 0.37</td></tr>
<tr><td></td><td>Brown</td><td>-1.70 | -1.79 || 0.63 | -1.71</td><td>0.62 | 0.77 || 0.62 | 0.84</td><td>-2.73 | -2.33 || 1.02 | -2.05</td><td>0.01 | 0.02 || 0.31 | 0.04</td></tr>
<tr><td></td><td>Green</td><td>-0.79 | -1.73 || 0.95 | -1.22</td><td>0.68 | 0.94 || 0.86 | 0.96</td><td>-1.16 | -1.84 || 1.10 | -1.27</td><td>0.25 | 0.07 || 0.27 | 0.20</td></tr>
<tr><td></td><td>Yellow</td><td>-1.32 | -1.64 || 1.31 | -1.46</td><td>0.77 | 0.96 || 0.65 | 1.04</td><td>-1.71 | -1.72 || 2.01 | -1.40</td><td>0.09 | 0.09 || 0.04 | 0.16</td></tr>
<tr><td></td><td>Blue</td><td>-2.38 | -2.25 || 1.55 | -1.80</td><td>1.13 | 1.27 || 0.82 | 1.32</td><td>-2.10 | -1.78 || 1.90 | -1.37</td><td>0.04 | 0.08 || 0.06 | 0.17</td></tr>
<tr><td></td><td>Orange</td><td>-0.66 | -0.26 || 1.93 | 0.11</td><td>1.64 | 1.77 || 2.08 | 2.01</td><td>-0.40 | -0.15 || 0.93 | 0.05</td><td>0.69 | 0.88 || 0.35 | 0.96</td></tr>
<tr><td></td><td>Purple</td><td>-0.51 | -0.04 || 1.67 | 0.11</td><td>1.72 | 1.99 || 1.90 | 2.01</td><td>-0.30 | -0.02 || 0.88 | 0.06</td><td>0.77 | 0.98 || 0.38 | 0.95</td></tr>
<tr><td></td><td>Black</td><td>-2.05 | -2.20 || 2.00 | -2.17</td><td>0.68 | 0.86 || 0.96 | 0.95</td><td>-3.01 | -2.57 || 2.09 | -1.30</td><td>0.00 | 0.01 || 0.04 | 0.02</td></tr>
<tr><td></td><td>Pink</td><td>-0.03 | / || 2.31 | /</td><td>2.77 | / || 2.62 | /</td><td>-0.01 | / || 0.88 | /</td><td>0.99 | / || 0.38 | /</td></tr>
<tr><td></td><td>Barred</td><td>-0.86 | -0.75 || 1.92 | -0.13</td><td>1.69 | 1.63 || 1.02 | 1.64</td><td>-0.51 | -0.46 ||1.88 | -0.08</td><td>0.61 | 0.65 || 0.06 | 0.94</td></tr>
<tr><td></td><td>Bald</td><td>1.95 | 1.44 || 5.56 | 1.80</td><td>0.80 | 0.97 || 2.08 | 1.05</td><td>2.43 | 1.49 || 2.67 | 1.72</td><td>0.02 | 0.14 || 0.01 | 0.09</td></tr>
<tr><td colspan="6">UV radiance male | female, complete tree || molecular tree only
n=1258, AIC=466.4, logLik=-230.2, R^2=0.11 | n=1262, AIC=455.9, logLik=-224.9, R^2=0.12 || n=1032, AIC=361.8, -logLik=-177.9, R^2=0.08 | n=1034, AIC=356.8, -logLik=-175.4, R^2=0.08</td></tr>
<tr><td></td><td>Intercept</td><td>-27.85 | -20.25 || -5.40 | -5.04</td><td>9.95 | 9.48 || 1.25 | 1.22</td><td>-2.79 | -2.14 || -4.32 | -4.12</td><td>0.01 |0.03 || 0.00 | 0.00</td></tr>
<tr><td></td><td>UV radiance</td><td>26.05 | 17.99 || 3.63 | 3.05</td><td>10.40 | 9.91 || 1.81 | 1.83</td><td>2.50 | 1.82 || 2.01 | 1.66</td><td>0.01 | 0.07 || 0.05 | 0.10</td></tr>
<tr><td colspan="6">Dichromatism male| female, complete tree || molecular tree only
n=1267, AIC=482.7, logLik=-238.4, R^2=0.09 | n=1266, AIC=458.8, logLik=-226.4, R^2=0.05 || n=1038, AIC=366.3, -logLik=-180.2, R^2=0.07 | n=1038, AIC= AIC=360.1, -logLik=-177.1, R^2=0.07</td></tr>
<tr><td></td><td>Intercept</td><td>-3.08 | -2.99 || -3.05 | -3.02</td><td>0.28 | 0.28 || 0.30 | 0.29</td><td>-10.96 | -10.67 || -10.10 | -10.48</td><td>0.00 | 0.00 || 0.00 | 0.00</td></tr>
<tr><td></td><td>Present</td><td>0.23 | -0.12 || 0.10 | -0.11</td><td>0.28 | 0.29 || 0.31 | 0.33</td><td>0.81 | -0.42 || 0.32 | -0.33</td><td>0.42 | 0.67 || 0.75 | 0.74</td></tr>
<tr><td colspan="6">Colonial male| female, complete tree || molecular tree only
n=1267, AIC=484.0, logLik=-239.0, R^2=0.09 | n=1267, AIC=459.1, logLik=-226.6, R^2=0.08 || n=1038, AIC=364.4, -logLik=-179.2, R^2=0.08 | n=1038, AIC=356.4, -logLik=-175.2, R^2=0.08</td></tr>
<tr><td></td><td>Intercept</td><td>-2.96 | -3.12 || -2.96 | -3.02</td><td>0.23 | 0.26 || 0.26 | 0.27</td><td>-12.61 | -12.03 || -11.50 | -11.24</td><td>0.00 | 0.00 || 0.00 | 0.00</td></tr>
<tr><td></td><td>Yes</td><td>0.10 | 0.30 || -0.64 | -0.83</td><td>0.41 | 0.41 || 0.60 | 0.67</td><td>0.25 | 0.72 || -1.07 | -1.24</td><td>0.80 | 0.47 || 0.29 | 0.22</td></tr>
<tr><td colspan="6">Closed habitat male| female, complete tree || molecular tree only
n=1267, AIC=483.9, logLik=-239, R^2=0.09 | n=1267, AIC=459.3, logLik=-226.7, R^2=0.03 || n=1038, AIC=366.5, -logLik=-180.2, R^2=0.07 | n=1038, AIC=360.0, -logLik=177.0, R^2=0.07</td></tr>
<tr><td></td><td>Intercept</td><td>-2.9 |-3.10 || -2.97 | -3.12</td><td>0.24 | 0.26 || 0.27 | 0.27</td><td>-12.19 | -11.70 || -11.01 | -11.67</td><td>0.00 | 0.00 || 0.00 | 0.00</td></tr>
<tr><td></td><td>Yes</td><td>0.06 | 0.09 || -0.07 | 0.25</td><td>0.26 | 0.27 || 0.30 | 0.37</td><td>0.24 | 0.34 || -0.22 | 0.67</td><td>0.81 | 0.73 || 0.83 | 0.50</td></tr>
<tr><td colspan="6">Age male| female, complete tree || molecular tree only
n=1207, AIC=458.7, logLik=-226.3, R^2=0.10 | n=1216, AIC=452, logLik=-223n R^2=0.04 || n=991, AIC=351.1, -logLik=-172.5, R^2=0.07 | n=1002, AIC=354.5, -logLik=-174.3, R^2=0.06</td></tr>
<tr><td></td><td>Intercept</td><td>0.07 | -2.86 || 2.31 | -2.73</td><td>5.93 | 0.86 || 6.74 | 2.57</td><td>0.01 | -3.33 || 0.34 | -1.06</td><td>0.99 | 0.00 || 0.73 | 0.29</td></tr>
<tr><td></td><td>Age</td><td>-29.68 | -1.18 || -5.53 | -0.45</td><td>58.48 | 8.09 || 7.05 | 3.86</td><td>-0.51 | -0.15 || -0.78 | -0.12</td><td>0.61 | 0.88 || 0.43 | 0.91</td></tr>
<tr><td colspan="6">Mass male| female, complete tree || molecular tree only
n=1184, AIC=415.6 , logLik=--204.8, R^2=0.10 | n=1181, AIC=448.4, logLik=-221.2, R^2=0.11 || n=1034, AIC=-365.9, -logLik=180.0, R^2=0.07 | n=992, AIC=351.4, -logLik=-172.7</td></tr>
</table>

<table>
<tr><td></td><td>Intercept</td><td>-4.69 | -3.19 || -3.16 | -2.33</td><td>0.79 | 0.31 || 0.38 | 0.58</td><td>-5.98 | -10.32 || -8.38 | -4.04</td><td>0.00 | 0.00 || 0.00 | 0.00</td></tr>
<tr><td></td><td>Mass</td><td>4.71 | 3.32 || 0.50 | 0.21</td><td>1.18 | 1.46 || 0.86 | 0.19</td><td>4.00 | 2.28 || 0.58 | 1.11</td><td>0.01 | 0.02 || 0.56 | 0.27</td></tr>
<tr><td colspan="6">Total precipitation male| female, complete tree || molecular tree only
n=1262, AIC=482.9, logLik=-238.5, R^2=0.09 | n=1262, AIC=458.5, logLik=-226.3, R^2=0.11 || n=1034, AIC=365.9, -logLik=-180.0, R^2=0.06 | n=1032, AIC=359.5, -logLik=-176.8, R^2=0.07</td></tr>
<tr><td></td><td>Intercept</td><td>-2.80 | -2.73 || -3.16 | -2.90</td><td>0.31 | 0.67 || 0.38 | 0.34</td><td>-9.01 | -4.09 || -8.38 | -8.55</td><td>0.00 | 0.00 || 0.00 | 0.00</td></tr>
<tr><td></td><td>Total precipitation</td><td>-0.51 | -0.49 || 0.50 | -0..63</td><td>0.76 | 1.27 || 0.86 | 0.85</td><td>-0.66 | -0.38 || 0.58 | -0.75</td><td>0.51 | 0.70 || 0.56 | 0.45</td></tr>
<tr><td colspan="6">Maximum temperature male| female, complete tree || molecular tree only
n=1262, AIC=471.3, logLik=-231.1, R^2=0.03 | n=1261, AIC=457.5, logLik=-225.8, R^2=0.03 || n=1034, AIC=391.1, -logLik=-192.6, R^2=0.00 | n=1032, AIC=368.3, -logLik=-181.1, R^2=0.04</td></tr>
<tr><td></td><td>Intercept</td><td>-4.18 | -4.07 || -4.35 | -1.55</td><td>0.73 | 0.73 || 1.13 | 0.38</td><td>-5.71 | -5.54 || -3.83 | -4.06</td><td>0.00 | 0.00 || 0.00 | 0.00</td></tr>
<tr><td></td><td>Max. Temp.</td><td>1.66 | 1.36 || 0.09 | -0.04</td><td>0.93 | 0.93 || 0.02 | 0.01</td><td>1.79 | 1.45 || 3.90 | -2.54</td><td>0.07 | 0.15 || 0.00 | 0.01</td></tr>
</table>

Supplementary Table 8. Relative occurrence of black skin in function of white feathers and baldness.

	male			female		
	white feathers	bald	no white feathers	white feathers	bald	no white feathers
black skin	30	17	72	25	22	81
non-black skin	47	16	1821	43	17	2056

Supplementary Table 9. : Extinct birds investigated. Black skinned species in bold.

Order	Family	Species
Anseriformes	Anatidae	*Rhodonessa caryophyllacea*
Anseriformes	Anatidae	*Camptorhynchus labradorius*
Gruiformes	Ralidae	*Nesoclopeus poecilopterus*
Charadriiformes	Alcidae	*Pinguinus impennis*
Strigiformes	Strigidae	*Sceloglaux albifacies*
Columbiformes	Columbidae	*Ectopistes migratoria*
Columbiformes	Columbidae	*Microgoura meeki*
Psittaciformes	Psittacidae	*Conuropsis carolinensis*
Passeriformes	Acanthisittidae	*Xenicus longipes*
Passeriformes	Oriolidae	*Turnagra capensis*
Passeriformes	Mohoidae	*Moho nobilis*
Passeriformes	Mohoidae	*Chaetoptila angustipluma*
Passeriformes	Fringillidae	*Chloridops kona*
Passeriformes	Fringillidae	*Rhodacanthis palmeri*
Passeriformes	Fringillidae	*Ciridops anna*
Passeriformes	Fringillidae	*Akialoa obscurus*
Passeriformes	Fringillidae	*Viridonia sagittirostris*
Passeriformes	Sturnidae	***Fregilupus varius***

Supplementary Table 10. Relative presence of black skin. Few species (e.g. *Casuaris*) that have black preserved skin had bright colouration on exposed skin in life. These bright colours were lost in preserved specimens (likely through dehydration and decay of colour-producing collagen fibers) but preserved as black skin.

Order	Family	Species	Proportion of sampled with black skin
Accipitriformes	Accipitridae	*Gyps indicus*	2/2
Accipitriformes	Accipitridae	*Pithecophaga jefferyi*	1/1
Anseriformes	Anatidae	*Anser caerulescens*	4/4
Anseriformes	Anatidae	*Coscoroba coscoroba*	1/1
Anseriformes	Anatidae	*Mergellus albellus*	1/2
Anseriformes	Anatidae	*Oressochen melanopterus*	1/1
Anseriformes	Anatidae	*Tachyeres pteneres* (ventral)	1/1
Apodiformes	Trochilidae	*Anopetia gounellei*	2/2

Bucerotiformes	Bucerotidae	*Rhyticeros plicatus*	1/2
Casuariiformes	Casuariidae	*Casuarius casuarius*	1/1
Cathartiformes	Carthartidae	*Coragyps atratus*	4/4
Cathartiformes	Carthartidae	*Vultur gryphus*	1/1
Charadriiformes	Alcidae	*Brachyramphus marmoratus*	2/2
Charadriiformes	Alcidae	*Cepphus carbo*	2/2
Charadriiformes	Dromadidae	*Dromas ardeola*	2/2
Charadriiformes	Jacanidae	*Actophilornis africanus*	10/10
Charadriiformes	Jacanidae	*Hydrophasianus chirurgus*	4/4
Charadriiformes	Jacanidae	*Microparra capensis*	1/1
Charadriiformes	Laridae	*Creagrus furcatus*	11/11
Charadriiformes	Laridae	*Larus fuscus*	6/8
Charadriiformes	Laridae	*Pagophila eburnea*	7/7
Charadriiformes	Laridae	*Leucophaeus modestus*	5/5
Charadriiformes	Recurvirostridae	*Cladorhynchus leucocephala*	2/2
Charadriiformes	Recurvirostridae	*Recurvirostra americana*	5/5
Ciconiiformes	Ciconiidae	*Jabiru mycteria*	1/1
Coliiformes	Coliidae	*Colius colius*	4/4
Coliiformes	Coliidae	*Colius leucocephalus*	5/5
Coliiformes	Coliidae	*Colius striatus*	1/5
Coliiformes	Coliidae	*Urocolius indicus*	5/5
Columbiformes	Columbiidae	*Alectroenas sganzini*	1/1
Galliformes	Megapodiidae	*Talegalla cuvieri*	2/2
Galliformes	Megapodiidae	*Macrocephalon maleo*	3/3
Galliformes	Numididae	*Acryllium vulturinum*	4/4
Galliformes	Numididae	*Guttera pucherani*	*10/10*
Galliformes	Numididae	*Numida meleagris*	2/2
Galliformes	Phasianidae	*Argusianus argus*	6/6
Galliformes	Phasianidae	*Meleagris ocellata*	2/2
Galliformes	Phasianidae	*Tragopan temminckii* (full body)	3/4
Gruiformes	Gruidae	*Bugeranus carunculatus*	1/1
Gruiformes	Rallidae	*Amaurornis marginalis*	7/10
Gruiformes	Ralidae	*Hapalocrex flaviventer*	2/2
Gruiformes	Ralidae	*Porphyriops melanops*	1/2
Musophagiformes	Musophagidae	*Corythaeola cristata*	10/10
Musophagiformes	Musophagidae	*Tauraco leucolophus*	10/10
Musophagiformes	Musophagidae	*Tauraco porphyreolophus*	1/2
Otidiformes	Otididae	*Ardeotis kori*	3/3
Opisthocomiformes	Opisthocomidae	*Opisthocomus hoazin*	10/10
Pelecaniformes	Ardeidae	*Ardea alba*	8/8
Pelecaniformes	Ardeidae	*Bubulcus ibis*	10/10
Pelecaniformes	Ardeidae	*Egretta thula*	5/5
Pelecaniformes	Threskiornithidae	*Theristicus caudatus*	4/4
Pelecaniformes	Ardeidae	*Egretta caerulea*	4/5
Pelecaniformes	Threskiornithidae	*Pseudibis papillosa*	2/2
Pelicaniformes	Threskiornithidae	*Threskiornis aethiopicus*	9/10
Phoenicopteriformes	Phoenicopteridae	*Phoenicoparrus andinus*	4/4
Phoenicopteriformes	Phoenicopteridae	*Phoenicopterus chilensis*	3/3
Piciformes	Capitonidae	*Capito niger*	2/2
Piciformes	Lybiidae	*Gymnobucco sladeni*	10/10
Piciformes	Lybiidae	*Lybius torquatus*	10/10
Piciformes	Picidae	*Mulleripicus fulvus*	2/2
Piciformes	Picidae	*Sapheopipo noguchii*	2/2
Podocipediformes	Podicipedidae	*Aechmophorus occidentalis* (full	2/2
Procellariiformes	Procellariidae	*Pterodroma neglecta* (full body)	1/1
Rheiformes	Rheidae	*Rhea americana*	3/3
Sphenisciformes	Spheniscidae	*Aptenodytes patagonicus* (ventral)	1/1
Sphenisciformes	Spheniscidae	*Megadyptes antipodes*	1/1
Suliformes	Sulidae	*Morus bassanus* (full body)	5/5
Suliformes	Sulidae	*Morus capensis* (full body)	2/2
Suliformes	Sulidae	*Papasula abbotti* (full body)	1/1

Phoenicopteriformes	Phoenicopteridae	*Phoeniconaias minor* (full body)	2/2
Passeriformes	Acanthizidae	*Calamanthus fuliginosus*	1/1
Passeriformes	Acanthizidae	*Hylacola cautus*	1/1
Passeriformes	Cardinalidae	*Incana incana*	8/8
Passeriformes	Certhiidae	*Salpornis spilonotus*	6/7
Passeriformes	Cisticolidae	*Spiloptila clamans*	5/5
Passeriformes	Cisticolidae	*Drymocichla incana*	9/9
Passeriformes	Cisticolidae	*Eremomela badiceps*	14/15
Passeriformes	Cisticolidae	*Malcorus pectoralis*	1/1
Passeriformes	Cisticolidae	*Neomixis tenella*	1/1
Passeriformes	Cisticolidae	*Prinia erythroptera*	2/2
Passeriformes	Cisticolidae	*Schistolais leucopogon*	10/10
Passeriformes	Cotingidae	*Carpodectes nitidus*	1/2
Passeriformes	Cotingidae	*Gymnoderus foetidus*	5/5
Passeriformes	Cotingidae	*Perissocephalus tricolor*	3/3
Passeriformes	Cotingidae	*Procnias nudicollis*	3/4
Passeriformes	Dicaeidae	*Dicaeum cruentatum*	5/5
Passeriformes	Estrildidae	*Estrilda perreini*	2/2
Passeriformes	Estrildidae	*Sporaeginthus subflavus*	5/10
Passeriformes	Fringillidae	*Telespiza cantans*	2/2
Passeriformes	Furnariidae	*Dendrexetastes rufigula*	2/2
Passeriformes	Furnariidae	*Hylexetastes perrotii*	1/1
Passeriformes	Hyliotidae	*Hyliota flavigaster*	5/5
Passeriformes	Icteridae	*Amblyramphus holosericeus*	5/5
Passeriformes	Laniidae	*Eurocephalus ruppelli*	7/8
Passeriformes	Leiothrichidae	*Ianthocincla albogularis*	1/2
Passeriformes	Macrosphenidae	*Pholidornis rushiae*	10/10
Passeriformes	Meliphagidae	*Conopophila rufogularis*	2/2
Passeriformes	Meliphagidae	*Entomyzon cyanotis*	3/4
Passeriformes	Meliphagidae	*Glycichaera fallax*	2/2
Passeriformes	Meliphagidae	*Sugomel niger*	2/2
Passeriformes	Menuridae	*Menura alberti*	1/1
Passeriformes	Nectariniidae	*Chalcomitra rubescens*	10/10
Passeriformes	Nectariniidae	*Deleornis fraseri*	13/15
Passeriformes	Nectariniidae	*Hedydipna platura*	10/10
Passeriformes	Nectariniidae	*Nectarinia kilimensis*	10/10
Passeriformes	Nectariniidae	*Anthreptes seimundi*	6/6
Passeriformes	Nicatoridae	*Nicator chloris*	10/10
Passeriformes	Paradisaeidae	*Manucodia ater*	5/6
Passeriformes	Paradisaeidae	*Seleucidis melanoleucus*	5/5
Passeriformes	Paradisaeidae	*Paradisaea apoda*	2/2
Passeriformes	Paradisaeidae	*Cicinnurus regius*	5/9
Passeriformes	Paradisaeidae	*Cicinnurus respublica*	8/8
Passeriformes	Paramythiidae	*Paramythia montium*	2/3
Passeriformes	Paridae	*Cephalopyrus flammiceps*	2/2
Passeriformes	Pellorneidae	*Malacopteron magnum*	4/4
Passeriformes	Philepittidae	*Neodrepanis coruscens*	1/2
Passeriformes	Picathartidae	*Picathartes gymnocephalus*	3/3
Passeriformes	Ploceidae	*Anaplectes rubriceps*	1/2
Passeriformes	Ploceidae	*Dinemellia dinemelli*	7/10
Passeriformes	Ploceidae	*Foudia eminentissima*	6/10
Passeriformes	Ploceidae	*Malimbus rubricollis*	10/10
Passeriformes	Ploceidae	*Pseudonigrita arnaudi*	5/5
Passeriformes	Pycnonotidae	*Arizelocichla masukuensis*	2/2
Passeriformes	Pycnonotidae	*Calyptocichla serinus*	6/6
Passeriformes	Pycnonotidae	*Hemixos flavala*	2/2
Passeriformes	Pycnonotidae	*Ixos mcclellandii*	1/1
Passeriformes	Pycnonotidae	*Neolestes torquatus*	6/10
Passeriformes	Remizidae	*Anthoscopus caroli*	7/7
Passeriformes	Remizidae	*Auriparus flaviceps*	4/6
Passeriformes	Sturnidae	*Agropsar sturninus*	1/2

Passeriformes	Sturnidae	*Leucopsar rothschildi*	1/1
Passeriformes	Sturnidae	*Neocichla gutturalis*	1/2
Passeriformes	Sturnidae	*Sturnornis albofrontatus*	5/5
Passeriformes	Thamnophilidae	*Gymnocichla nudiceps*	2/2
Passeriformes	Tyrannidae	*Camptostoma obsoletum*	2/2
Passeriformes	Tyrannidae	*Deltarhynchus flammulatus*	2/2
Passeriformes	Tyrannidae	*Ornithion semiflavum*	2/2
Passeriformes	Tyrannidae	*Pseudelaenia leucospodia*	8/8
Passeriformes	Vangidae	*Falculea palliata*	4/4
Passeriformes	Vangidae	*Vanga curvirostris*	3/3
Passeriformes	Vangidae	*Artamella viridis*	2/2

Supplementary Table 11. Alcohol preserved specimens examined. Species names in bold represent species that had black skin. Congruence between alcohol preservation and preserved as dried skin is indicated by the absence of a superscript when the same species was compared, or with a “+” in superscript if comparison was between congeneric species. Discordance between preservation methods is marked with a “-“ in superscript, or with a “*” if comparison was between congeneric species.

Order	Family	Species
Anseriformes	Anatidae	*Dendrocygna viduata*
Bucerotiformes	Bucerotidae	***Bycaniste fistulator***$^{-}$
Charadriiformes	Glareolidae	*Cursorius cursor*
Charadriiformes	Jacanidae	***Actophilornis africana***
Charadriiformes	Laridae	*Rhynchops flavirostris*
Charadriiformes	Laridae	*Sterna hirundo*$^{+}$
Charadriiformes	Laridae	*Sterna nigra*$^{+}$
Galliformes	Numididae	***Acryllium vulturinum***
Galliformes	Numididae	*Guttera plumifera*$^{-}$
Galliformes	Numididae	***Numida melagris***
Musophagiformes	Musophagidae	*Corythaixides personata*$^{+}$
Musophagiformes	Musophagidae	*Musophaga violacea*$^{+}$
Musophagiformes	Musophagidae	***Corythaeola cristata***
Musophagiformes	Musophagidae	***Tauraco persa***$^{+}$
Musophagiformes	Musophagidae	***Tauraco schutti***$^{+}$
Otidiformes	Otididae	*Neotis denhami*
Pelecaniformes	Ardeidae	***Ardea alba***
Pelecaniformes	Ardeidae	*Ardea purpurea*
Pelecaniformes	Ardeidae	***Ardeola ralloides***$^{-}$
Pelecaniformes	Ardeidae	***Bubulcus ibis***
Pelecaniformes	Ardeidae	***Egretta garzetta***$^{+}$
Pelecaniformes	Ardeidae	*Nycticorax nycticorax*
Pelicaniformes	Threskiornithidae	***Threskiornis aethiopicus***
Phoenicopteriformes	Phoenicopteridae	***Phoeniconaias minor***
Piciformes	Lybiidae	*Gymnobucco bonapartei**
Piciformes	Lybiidae	*Pogoniulus chrysoconus*$^{+}$
Podicipediformes	Podicipedidae	*Podiceps ruficollis*
Sphenisciformes	Spheniscidae	*Spheniscus* sp. $^{+}$
Strigiformes	Coliidae	***Urocolius macrourus***
Suliformes	Phalacrocoracidae	Phalacrocorax africanus
Suliformes	Sulidae	***Morus capensis***
Passeriformes	Estrildidae	***Estrilda caerulescens*** $^{+}$
Passeriformes	Hyliotidae	***Hyliota falvigaster***
Passeriformes	Muscicapidae	*Alethe diademata*
Passeriformes	Muscicapidae	***Pogonocichla stellata***$^{-}$
Passeriformes	Nectariniidae	***Nectarinia kilimensis***
Passeriformes	Nicatoridae	*Andropadus virens +*
Passeriformes	Oriolidae	*Oriolius larvatus* $^{+}$
Passeriformes	Ploceidae	*Euplectes ardens*
Passeriformes	Ploceidae	*Foudia eminentissima*$^{-}$
Passeriformes	Ploceidae	***Malimbus rubricollis***
Passeriformes	Pycnonotidae	***Andropadus curvirostris ****
Passeriformes	Pycnonotidae	*Baeopogon indicator*
Passeriformes	Pycnonotidae	*Ixonotus guttatus*
Passeriformes	Pycnonotidae	*Phyllastrephus albigularis* $^{+}$
Passeriformes	Pycnonotidae	*Pycnonotus barbatus*
Passeriformes	Pycnonotidae	*Thescelocichla leucopleura*
Passeriformes	Remizidae	*Remiz caroli*$^{-}$
Passeriformes	Viduidae	***Anomalospiza imberbis***$^{-}$

Supplementary Table 12. Live specimens examined.

Order	Family	Species	Skin color
Passeriformes	Parulidae	*Geothlypis trichas*	red
Passeriformes	Parulidae	*Setophaga castanea*	red
Passeriformes	Parulidae	*Setophaga striata*	red
Passeriformes	Corvidae	*Cyanocitta cristata*	red
Passeriformes	Turdidae	*Catharus ustulatus*	red
Passeriformes	Parulidae	*Setophaga pinus*	red
Passeriformes	Vireonidae	*Vireo olivaceus*	red
Passeriformes	Troglodytidae	*Troglodytes aedon*	red
Passeriformes	Paridae	*Poecile atricapillus*	red
Passeriformes	Troglodytidae	*Thryothorus ludovicianus*	red
Passeriformes	Mimidae	*Dumetella carolinensis*	red
Passeriformes	Mimidae	*Toxostoma rufum*	red
Suliformes	Sulidae	*Morus bassanus*	black
Passeriformes	Corvidae	*Garrulus glandarius*	red
Charadriiformes	Laridae	*Larus fuscus*	red

Supplementary Table 13. Mismatches between our colour assignment and that of HBW for feathers colours on the back of the head. Rows correspond to colours assigned in this manuscript, columns to colours assigned by HBW. Numbers correspond to the number of times that colours were differently assigned. The colour scale corresponds to the degree of mismatch.

		**Colour assignment**											
		barred	black	white	brown	grey	orange	blue	green	purple	red	yellow	pink
**HBW**	barred	0	0	0	2	0	0	0	0	0	0	0	0
	black	0	0	2	56	13	0	6	0	1	2	1	0
	white	1	1	0	4	2	0	0	0	0	0	0	0
	brown	2	27	2	0	15	3	0	7	0	6	9	0
	grey	0	20	1	108	0	0	2	2	0	0	0	1
	orange	0	0	0	0	0	0	0	0	0	0	0	0
	blue	0	16	0	2	3	1	0	4	4	0	0	0
	green	0	8	0	33	12	0	0	0	0	0	33	0
	purple	0	3	0	3	0	0	0	0	0	0	0	0
	red	0	0	0	6	1	0	0	0	2	0	1	0
	yellow	0	1	0	9	0	1	1	2	0	0	0	0
	pink	0	0	0	0	0	0	0	0	0	0	0	0
