# Supplementary material for: Exposure to UV radiance predicts repeated evolution of concealed black skin in birds
Source: Nat Commun. 2020 May 15;11:2414. doi: 10.1038/s41467-020-15894-6 (PMC7229023; doi:10.1038/s41467-020-15894-6)
Supplement: Supplementary file 2 — Reporting Summary [file 41467_2020_15894_MOESM2_ESM.pdf]

nature research

Corresponding author(s): DBPR NCOMMS-19-28791

Last updated by author(s): 7/12/2019

Reporting Summary

Nature Research wishes to improve the reproducibility of the work that we publish. This form provides structure for consistency and transparency in reporting. For further information on Nature Research policies, seeAuthors & Referees and theEditorial Policy Checklist .

Statistics

For all statistical analyses, confirm that the following items are present in the figure legend, table legend, main text, or Methods section.

n/a	Confirmed	
☐	☒	The exact sample size (*n*) for each experimental group/condition, given as a discrete number and unit of measurement
☐	☒	A statement on whether measurements were taken from distinct samples or whether the same sample was measured repeatedly
☐	☒	The statistical test(s) used AND whether they are one- or two-sided<br>*Only common tests should be described solely by name; describe more complex techniques in the Methods section.*
☐	☒	A description of all covariates tested
☐	☒	A description of any assumptions or corrections, such as tests of normality and adjustment for multiple comparisons
☐	☒	A full description of the statistical parameters including central tendency (e.g. means) or other basic estimates (e.g. regression coefficient) AND variation (e.g. standard deviation) or associated estimates of uncertainty (e.g. confidence intervals)
☐	☒	For null hypothesis testing, the test statistic (e.g. *F*, *t*, *r*) with confidence intervals, effect sizes, degrees of freedom and *P* value noted<br>*Give P values as exact values whenever suitable.*
☒	☐	For Bayesian analysis, information on the choice of priors and Markov chain Monte Carlo settings
☒	☐	For hierarchical and complex designs, identification of the appropriate level for tests and full reporting of outcomes
☐	☒	Estimates of effect sizes (e.g. Cohen's *d*, Pearson's *r*), indicating how they were calculated

Our web collection on statistics for biologists contains articles on many of the points above.

Software and code

Policy information about availability of computer code

Data collection	No software was used for data collection.
Data analysis	R 3.4.2 was used for all analyses in this project. Within R, analyses employ standard R packages.

For manuscripts utilizing custom algorithms or software that are central to the research but not yet described in published literature, software must be made available to editors/reviewers. We strongly encourage code deposition in a community repository (e.g. GitHub). See the Nature Research guidelines for submitting code & software for further information.

Data

Policy information about availability of data

All manuscripts must include a data availability statement. This statement should provide the following information, where applicable:

- Accession codes, unique identifiers, or web links for publicly available datasets
- A list of figures that have associated raw data
- A description of any restrictions on data availability

All data is available as Supplementary Data 1.

Field-specific reporting

Please select the one below that is the best fit for your research. If you are not sure, read the appropriate sections before making your selection.

☐ Life sciences ☐ Behavioural & social sciences ☒ Ecological, evolutionary & environmental sciences

For a reference copy of the document with all sections, see nature.com/documents/nr-reporting-summary-flat.pdf

Ecological, evolutionary & environmental sciences study design

All studies must disclose on these points even when the disclosure is negative.

Study description	Comparative analyses were used to look for associations between black skin and ecological/climatological variables. Analyses were corrected for phylogenetic dependency.
Research sample	2247 species from all families and >99% of bird genera studied in multiple natural history museums. Sample size was planned to encompass genus-level diversity. The limiting factor that prevented us from looking at all bird genera was the availability of specimens at the museums visited. If available, data of both sexes was collected to include the possible presence of sexual dimorphism. We collected data from adult specimens when possible to control for potential ontogenetic effects. For six species we only had juvenile specimens available. For nine species life stage was unavailable.
Sampling strategy	No sample-size calculations were done. Given the large and exploratory scope of the study we looked at one male and one female specimen assuming as a trade-off between obtaining as much data as possible but also limiting the number of specimens handled in often already small sample sizes available in the collections. If black skin was observed we looked at all specimens available with a maximum of 10 individuals per species.
Data collection	Data was collected by M.N. with help from S.P. and R.C. Feathers were gently lifted from the specimens to reveal skin. In small birds forceps were used. The presence/absence of black skin was noted. Distinction between black and non-black was obvious. Colour data for Eutrichomyas rowleyi and Xenoperdus obscuratus were by provided by Martin Päckert (Naturmuseum Senckenberg) and Jon Fjeldså (Natural History Museum of Denmark). Ecological data were obtained from Handbook of the birds of the world and IUCN. Climatic data were obtained from NASA Langley Research Center Power Project, Worldclim and gIUV. Distribution data was obtained from Global Biodiversity Information Facility.
Timing and spatial scale	Data was collected between june and september 2018. Specimens cover the full range of bird distribution.
Data exclusions	No data were excluded from the analyses.
Reproducibility	All data to reproduce the results are made available (Supplementary data 1). Analyses were redone using a second colour assignement for feather colour (see supplementary note 1). When black skin was observed we repeated the procedure for additional specimens. These results are shown in supplementary table 11.
Randomization	We were limited by the availability of specimens from the museum collection. As such randomization was not possible.
Blinding	Specimens need to be actively requested from curators and examined, as such blinding was not possible.

Did the study involve field work? ☐ Yes ☒ No

Reporting for specific materials, systems and methods

We require information from authors about some types of materials, experimental systems and methods used in many studies. Here, indicate whether each material, system or method listed is relevant to your study. If you are not sure if a list item applies to your research, read the appropriate section before selecting a response.

Materials & experimental systems

n/a	Involved in the study
☒	☐ Antibodies
☒	☐ Eukaryotic cell lines
☒	☐ Palaeontology
☐	☒ Animals and other organisms
☒	☐ Human research participants
☒	☐ Clinical data

Methods

n/a	Involved in the study
☒	☐ ChIP-seq
☒	☐ Flow cytometry
☒	☐ MRI-based neuroimaging

Animals and other organisms

Policy information about studies involving animals; ARRIVE guidelines recommended for reporting animal research

Laboratory animals	No laboratory animals were used in the study.
Wild animals	The crux of the study is based on museum specimens. Few observations on wild animals were made during open ringing-sessions by Mass Audubon and Manomet. These ringing sessions were done independently from this research.
Field-collected samples	No field collected samples were used in the study.

Ethics oversight	No information requiring ethical approval was involved in the study.

Note that full information on the approval of the study protocol must also be provided in the manuscript.
