# Supplementary material for: Exposure to UV radiance predicts repeated evolution of concealed black skin in birds
Source: Nat Commun. 2020 May 15;11:2414. doi: 10.1038/s41467-020-15894-6 (PMC7229023; doi:10.1038/s41467-020-15894-6)
Supplement: Supplementary file 3 — Description of Additional Supplementary Files [file 41467_2020_15894_MOESM3_ESM.pdf]

Description of Additional Supplementary Files

File Name: Supplementary Data 1
Description: Data on each sample used for analyses include, the collection of origin, the tag-number, species name, order and family and their representative age (whether adult or juvenile), sex, location sampled, collection and the (minimum) age of the specimen. Colour variables include feather colour, plumage colour as assigned by Handbook of the Birds of the World, skin colour, whether the sample is bald, presence/absence of sexual dimorphism and sexual dichromatism. Ecological variables include colonial lifestyle, IUCN habitats inhabited, mass, mean breeding latitude, mean breeding longitude and UV irradiance.
